# Variations in Circulating Tumor Microenvironment-Associated Proteins in Non-Muscle Invasive Bladder Cancer Induced by Mitomycin C Treatment

**DOI:** 10.3390/ijms26157413

**Published:** 2025-08-01

**Authors:** Benito Blanco Gómez, Francisco Javier Casas-Nebra, Daniel Pérez-Fentes, Susana B. Bravo, Laura Rodríguez-Silva, Cristina Núñez

**Affiliations:** 1 Urology Division, Lucus Augusti University Hospital (HULA), Servizo Galego de Saúde (SERGAS), 27002 Lugo, Spain; vvlanco@hotmail.com (B.B.G.); francisco.javier.casas.nebra@sergas.es (F.J.C.-N.); 2 Urology Division, University Clinical Hospital of Santiago de Compostela (CHUS), Servizo Galego de Saúde (SERGAS), 15706 Santiago de Compostela, Spain; daniel.adolfo.perez.fentes@sergas.es; 3 Proteomic Unit, Health Research Institute of Santiago de Compostela (IDIS), University Clinical Hospital of Santiago de Compostela (CHUS), 15706 Santiago de Compostela, Spain; sbbravo@gmail.com; 4 Inorganic Chemistry Department, Faculty of Sciences, Campus Terra, University of Santiago de Compostela, 27002 Lugo, Spain; laura.rodriguez@usc.es; 5 Research Unit, Hospital Universitario Lucus Augusti (HULA), Servizo Galego de Saúde (SERGAS), 27002 Lugo, Spain

**Keywords:** non-muscle invasive bladder cancer (NMIBC), mitomycin C (MMC), tumor microenvironment (TME), complement system, protein corona (PC), platinum nanoparticles (PtNPs), SWATH-MS

## Abstract

Mitomycin C (MMC) is a widely employed chemotherapeutic agent, particularly in non-muscle invasive bladder cancer (NMIBC), where it functions by inducing DNA cross-linking and promoting tumor cell apoptosis. However, the tumor microenvironment (TME) significantly influences the therapeutic efficacy of MMC. Among the key regulators within the TME, the complement system and the coagulation pathway play a crucial role in modulating immune responses to cancer therapies, including MMC. This article explores the interaction between platinum nanoparticles (PtNPs) with human serum (HS) of NMIBC patients (T1 and Ta subtypes) at three different points: before the chemotherapy instillation of MMC (*t*_0_) and three (*t*_3_) and six months (*t*_6_) after the treatment with MMC. This novel nanoproteomic strategy allowed the identification of a TME proteomic signature associated with the response to MMC treatment. Importantly, two proteins involved in the immune response were found to be deregulated across all patients (T1 and Ta subtypes) during MMC treatment: prothrombin (F2) downregulated and complement component C7 (C7) upregulated. By understanding how these biomarker proteins interact with MMC treatment, novel therapeutic strategies can be developed to enhance treatment outcomes and overcome resistance in NMIBC.

## 1. Introduction

Bladder cancer (BC) ranks among the top ten most frequently diagnosed cancers on a global scale, with approximately 573,000 new cases identified each year and an annual mortality of around 213,000 [1]. NMIBC comprises the majority of cases and is categorized into Ta, T1, and carcinoma in situ (CIS) stages based on the tumor’s depth of invasion [2]. Although NMIBC has a lower mortality risk compared to muscle-invasive bladder cancer (MIBC), its high recurrence and progression rates necessitate rigorous surveillance and intervention strategies [3,4].

MMC is a widely used chemotherapeutic agent, particularly in the treatment of NMIBC, where it is typically administered intravesically [5]. MMC functions by inducing DNA cross-linking, resulting in cell cycle arrest and tumor cell apoptosis [6]. Despite its effectiveness, the response to MMC can be variable, and the TME plays a critical role in determining the therapeutic outcomes.

Among the various components of the TME, the complement system has emerged as an influential factor in regulating the immune response to cancer therapies, modulating tumor cell sensitivity to DNA-damaging agents like mitomycin C (MMC) [7]. Importantly, recent studies suggest that the complement system’s activation can modulate the efficacy of MMC in BC [8].

Proteomics approaches have significantly advanced our understanding of the TME by enabling the comprehensive characterization of proteins involved in tumor progression, immune evasion, and therapy resistance [9,10,11,12,13]. Particularly, sequential window acquisition of all theoretical fragment ion mass spectra (SWATH-MS) has emerged as a powerful mass spectrometry (MS) technique for the comprehensive and reproducible analysis of circulating proteins derived from the TME. Unlike traditional data-dependent acquisition (DDA) methods, sequential window acquisition of all theoretical mass spectra (SWATH-MS) enables data-independent acquisition (DIA), allowing for the quantification of thousands of proteins across multiple samples with high reproducibility and depth [14]. This approach is particularly advantageous for analyzing circulating tumor-derived proteins, such as cytokines, growth factors, and extracellular vesicle-associated proteins, which play crucial roles in tumor progression, immune modulation, and metastasis [15]. Recent studies have demonstrated that SWATH-MS can accurately profile TME-associated circulating proteins in plasma and serum, facilitating the identification of non-invasive biomarkers for cancer diagnosis and treatment response monitoring [16,17,18,19]. As advancements in DIA-based proteomics continue, SWATH-MS is expected to further revolutionize the study of the TME and its systemic effects on the host.

Biological samples exhibit a high level of complexity, with protein concentrations spanning a broad dynamic range, thereby posing significant challenges to proteomic analysis [20]. To reduce sample complexity, fractionation or enrichment of specific cellular compartments can be performed prior to MS analysis.

The integration of nanomaterials into proteomics has led to the emergence of nanoproteomics, a rapidly growing research field [21]. It is well established that when nanomaterials are dispersed in physiological fluids, they spontaneously interact with proteins, forming a dynamic layer known as the PC. Notably, disease-related biomarkers constitute less than 1% of the total serum protein content, making their detection particularly challenging. Due to their high surface area and tunable physicochemical properties, nanoparticles (NPs) can function as selective sorbents, preferentially binding low-abundance proteins in serum samples, thereby facilitating biomarker enrichment prior to MS-based analysis [22,23,24,25,26,27,28]. The characterization of the PC surrounding NPs offers significant advantages over conventional proteomic approaches, enhancing the likelihood of discovering novel molecular biomarkers [29].

Particularly, the unique physicochemical properties of PtNPs [30] render them effective sorbent nanomaterials with significant potential for biomedical applications. Our previous studies provide robust protocols for the in vitro formation of the PC around PtNPs (2.40 ± 0.30 nm) following their interaction with HS [22]. The combined approach using PtNPs, electrophoretic separation (SDS-PAGE), and liquid chromatography–tandem mass spectrometry (LC-MS/MS) has proven effective in discovering novel circulating proteins linked to therapeutic response and resistance in HER2-positive breast cancer undergoing neoadjuvant chemotherapy [28]. In a comparable approach, our research group utilized silver nanoparticles (AgNPs; 9.73 ± 1.70 nm) as nanoscale scavenging platforms in conjunction with SWATH-MS to perform a comprehensive quantitative analysis of serum proteome alterations in two NMIBC subtypes: T1 and Ta. The ex vivo characterization of the protein coronas (PCs) formed on AgNPs facilitated the discrimination of two principal classes of serum proteins exhibiting differential abundance in NMIBC patients relative to healthy controls: components of the complement and coagulation cascades and various apolipoproteins [27].

**Figure 1:**
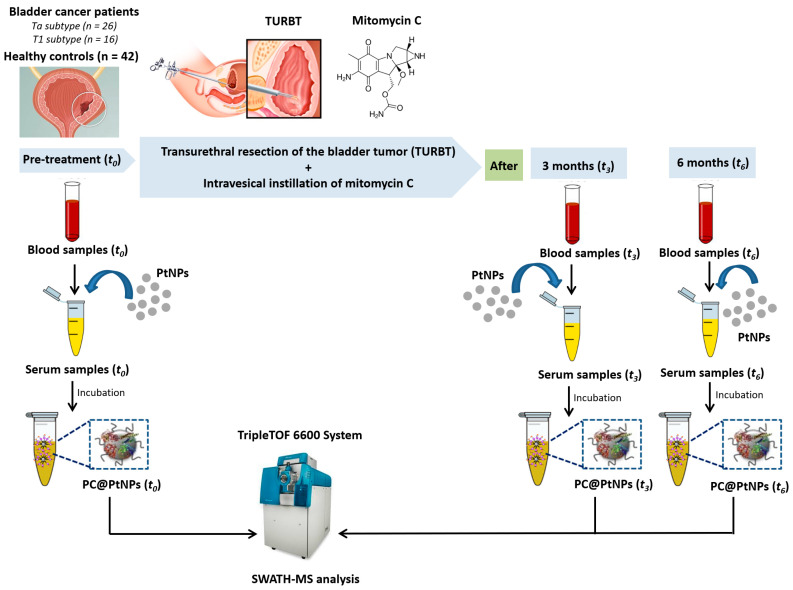
Diagrammatic overview of the protocol for PC assembly on PtNPs (2.40 ± 0.30 nm), following ex vivo incubation with HS from *n* = 42 healthy controls (HCs) and *n* = 42 NMIBC patients. Samples were collected at baseline (*t*_0_, pre-treatment) and three (*t*_3_) and six months (*t*_6_) post-mitomycin C (MMC) instillation.

Due to colloidal stability, their high surface-area-to-volume ratio, and their ability to conjugate with biomolecules [30], in the present work, PtNPs (2.40 ± 0.30 nm) were incubated with serum samples of NMIBC patients (*n* = 42) and healthy controls (*n* = 42) before the chemotherapy instillation of MMC (*t*_0_) and three (*t*_3_) and six months (*t*_6_) after treatment. Subsequently, an in-depth quantitative analysis of the PCs was performed using SWATH-MS to identify novel molecular targets associated with the distinct intrinsic subtypes of NMIBC, as well as for the identification of a TME proteomic signature associated with the MMC response (see Figure 1).

## 2. Results

### 2.1. Incubation of PtNPs (2.40 ± 0.30 nm) with HS Samples: Ex Vivo Formation and Comprehensive Characterization of the PC

HS samples from *n* = 42 HC and *n* = 42 NMIBC patients (*n* = 26 with the Ta subtype and *n* = 16 with the T1 subtype) were collected at times *t*_0_, *t*_3_, and *t*_6_ and processed and analyzed in the same manner (see Section 4.1).

**Figure 2:**
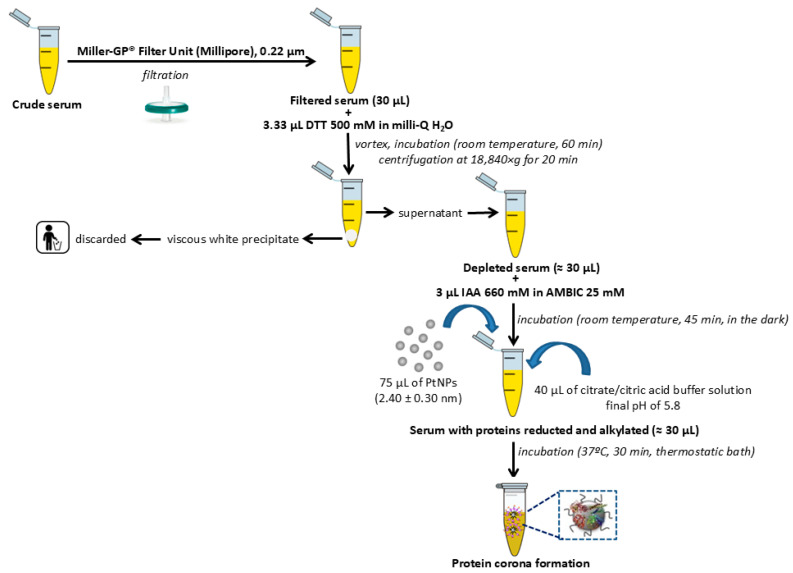
Flowchart depicting the general pretreatment, depletion with DDT, and alkylation with IAA of human serum samples before the incubation with PtNPs (2.40 ± 0.30 nm) for the PC formation.

PtNPs with a size of 2.40 ± 0.30 nm were prepared by a chemical reduction method [22,28,31] (see Section 4.3, Table S1). Proteins present in triplicate serum samples were first reduced using dithiothreitol (DTT) and subsequently alkylated with iodoacetamide (IAA). Following these modifications, the proteins were incubated with PtNPs averaging 2.40 ± 0.30 nm in diameter to facilitate the formation of PCs (see Figure 2) [22,28].

PtNPs were incubated separately with pooled HS samples obtained from *n* = 42 HC and *n* = 42 patients diagnosed with NMIBC. Following incubation, the PC-coated PtNPs were isolated by centrifugation and subsequently analyzed using transmission electron microscopy (TEM) and dynamic light scattering (DLS). Adsorption of serum proteins onto the nanoparticle surfaces induced a measurable increase in hydrodynamic diameter, with average sizes shifting from 2.40 ± 0.30 nm (bare PtNPs) to 2.55 ± 0.17 nm in the HC group and 2.65 ± 0.18 nm in the NMIBC group.

The association of cationic proteins with the surface of PtNPs is likely responsible for the observed shift in zeta potential, with the initially highly negative charge of −38.7 mV for unmodified PtNPs becoming less negative upon protein adsorption, reaching −25.3 mV in the HC condition and −27.2 mV in the NMIBC context [32,33].

### 2.2. Quantitative Analysis of the Protein Corona-Coated PtNPs by SWATH-MS Before the Chemotherapy Instillation of MMC (t_0_) and Three (t_3_) and Six Months (t_6_) After Treatment

Proteins bound to PtNPs were isolated using Sodium Dodecyl Sulfate Polyacrylamide Gel Electrophoresis (SDS-PAGE) and subsequently enzymatically digested according to established protocols [22,28]. The generated peptides were then subjected to quantitative analysis employing the advanced label-free proteomics technique, SWATH-MS.

The comparison of the ex vivo PC profiles enabled the identification of proteins exhibiting differential expression between HC and the two NMIBC subtypes (Ta and T1) across multiple time points (*t*_0_, *t*_3_, and *t*_6_). All analyses were subjected to statistical filtering, retaining only those proteins with a significance threshold of *p*-value ≤ 0.05.

### 2.3. Differentially Expressed Proteins in the Blood Serum of Control Patients and NMIBC Patients Before the Chemotherapy Instillation of MMC (t_0_)

At point *t*_0_, a total of 39 proteins were identified as differentially expressed in NMIBC patients with the T1 subtype, including 25 upregulated and 14 downregulated proteins. In contrast, 62 proteins were differentially expressed in patients with the Ta subtype, comprising 53 upregulated and 9 downregulated proteins (see Table 1). Comprehensive lists of candidate protein biomarkers exhibiting upregulation or downregulation in both NMIBC subtypes relative to healthy controls, along with the corresponding fold-change values, are provided in Tables S2 and S5.

**Figure 3:**
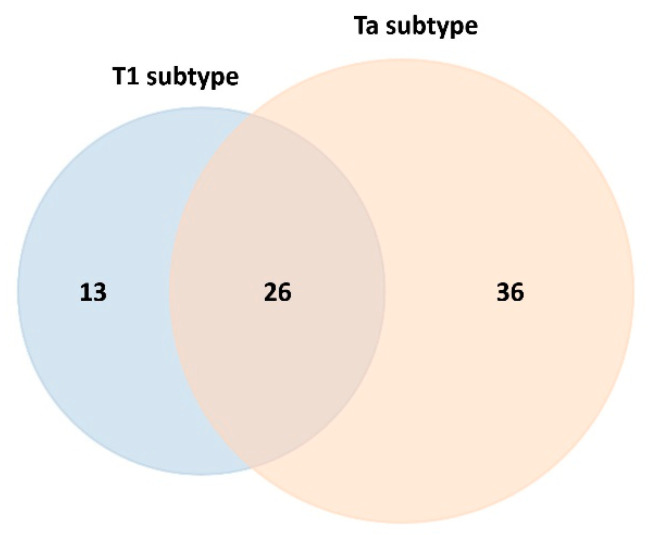
Venn diagram depicting the overlap and subtype-specific differentially expressed serum proteins adsorbed onto PtNPs (2.40 ± 0.30 nm) after 30 min ex vivo incubation with HS from NMIBC patients of subtypes T1 and Ta (data for the T1 subtype are highlighted in blue color and for the Ta subtype in orange).

The Venn diagram depicting statistically significant upregulated and downregulated proteins reveals that 26 proteins are commonly altered in both the T1 and Ta NMIBC subtypes (see Figure 3). Notably, 21 of these 26 proteins were consistently upregulated across both subtypes: alpha-1-antichymotrypsin (SERPINA3), alpha-1-antitrypsin (SERPINA1), alpha-1B-glycoprotein (A1BG), biotinidase (BTD), coagulation factor IX (F9), coagulation factor XII (F12), complement C4-B (C4B), complement component C7 (C7), complement component C8 alpha chain (C8A), complement component C9 (C9), galectin-3-binding protein (LGALS3BP), immunoglobulin alpha-2 heavy chain, immunoglobulin delta heavy chain, immunoglobulin heavy constant gamma 3 (IGHG3), immunoglobulin kappa variable 1-33 (IGKV1-33), lumican (LUM), monocyte differentiation antigen CD14 (CD14), plasminogen (PLG), platelet glycoprotein Ib alpha chain (GP1BA), serum amyloid A-4 protein (SAA4), and testis-expressed protein 33 (CIMIP4). On the other hand, 5 of these 26 proteins were found to be downregulated in the T1 and Ta NMIBC subtypes: alpha-2-HS-glycoprotein (AHSG), apolipoprotein M (APOM), carboxypeptidase N subunit 2 (CPN2), prothrombin (F2), and serum paraoxonase/arylesterase 1 (PON1) (see Tables S2 and S5).

SWATH-MS analysis further enabled the identification of subtype-specific proteins (see Figure 3), with 13 proteins uniquely associated with the T1 subtype (4 upregulated and 9 downregulated) and 36 proteins specific to the Ta subtype (32 upregulated and 4 downregulated), as detailed in Table 2 and Table 3, respectively.

### 2.4. The Biological Role of the NMIBC-Related Proteins Identified in the PtNP–Protein Corona Before the Chemotherapy Instillation of MMC (t_0_)

To analyze the global alterations in the serum proteome associated with NMIBC at baseline (*t*_0_), the STRING database was used to perform protein–protein interaction (PPI) network analysis. This approach enabled the identification of 26 commonly dysregulated proteins shared between the T1 and Ta subtypes, which exhibited significant differential expression between HC and NMIBC patients. Additionally, subtype-specific analyses revealed 13 proteins uniquely altered in the T1 subtype and 36 in the Ta subtype.

The analysis revealed that the dysregulated serum proteins are mainly involved in the immune response pathway. From the 26 proteins found to be commonly deregulated in both NMIBC subtypes, 8 proteins were implicated in the immune response pathway: coagulation factor XII (F12), complement C4-B (C4B), complement component C7 (C7), complement component C8 alpha chain (C8A), complement component C9 (C9), immunoglobulin kappa variable 1-33 (IGKV1-33), monocyte differentiation antigen CD14 (CD14), and prothrombin (F2). From these, C4B, C7, C8A, C9, F2, and F12 also participate in the complement and coagulation cascades pathways.

Particularly, 5 of the 13 biomarker proteins from the T1 subtype are involved in the immune response pathway, such as complement C4-A (C4A), C4b-binding protein beta chain (C4BPB), complement factor D (CFD), kininogen-1 (KNG1), and platelet basic protein (PPBP). From these, C4B, C4BPB, CFC, and KNG1 also participate in the complement and coagulation cascade pathways.

In the case of the Ta subtype, 15 from the 36 biomarker proteins are vinculated with the immune response pathway: N-acetylmuramoyl-L-alanine amidasa (PGLYRP2), complement C1q subcomponent subunit A (C1QA), complement C1r subcomponent (C1R), complement C1s subcomponent (C1S), C4b-binding protein alpha chain (C4BPA), complement component C6 (C6), complement component C8 beta chain (C8B), complement component C8 gamma chain (C8G), complement factor H (CFH), complement factor I (CFI), clusterin (CLU), ficolin-2 (FCN2), fibrinogen alpha chain (FGA), immunoglobulin heavy variable 4-38-2 (IGHV4-38-2 or LOC102723407), and plasma protease C1 inhibitor (SERPING1). From these, C1QA, C1R, C1S, C4BPA, C6, C8B, C8G, CFH, CFI, CLU, FGA, and SERPING1 are also involved in the complement and coagulation cascade pathways.

### 2.5. Comparison of the Differentially Expressed Proteins in the Blood Serum of Control and NMIBC Patients Before Treatment (t_0_) and Three (t_1_) and Six Months (t_2_) After the Chemotherapy Instillation of MMC

**Figure 4:**
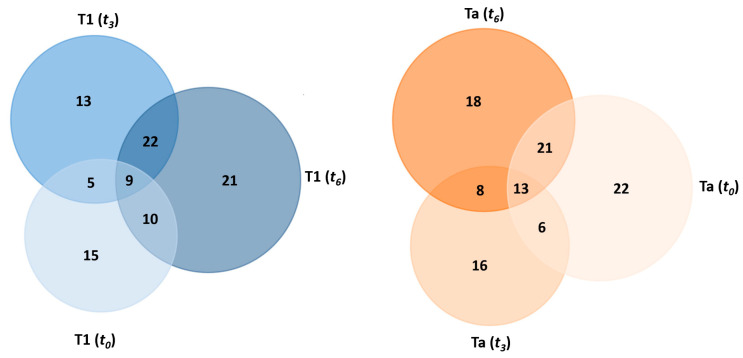
Venn diagram showing the number of shared and specific deregulated proteins identified in the PC-coated PtNPs (2.40 ± 0.30 nm) after their incubation (30 min) with HS samples from NMIBC patients of the T1 subtype (**left**) and Ta subtype (**right**) at different times (*t*_0_, *t*_3_, and *t*_6_).

As presented in Table 4, a total of 39, 49, and 62 proteins were found to be differentially expressed by liquid chromatography–tandem mass spectrometry (LC-MS/MS) in all serum samples from NMIBC patients with the T1 subtype, whereas 62, 43, and 60 proteins were identified in all serum samples from patients with the Ta subtype at time points *t*_0_, *t*_3_, and *t*_6_, respectively (see Table 4 and Tables S2–S7). A comparative analysis of the results obtained across the three points revealed that 9 and 13 proteins were commonly identified in T1 and Ta cases, respectively (see Table 4 and Figure 4).

Among the nine proteins commonly identified in the T1 subtype across all time points, four were consistently downregulated in all cases: insulin-like growth factor-binding protein complex acid-labile subunit (IGFALS), carboxypeptidase N subunit 2 (CPN2), prothrombin (F2), and apolipoprotein M (APOM). Three proteins were consistently upregulated: complement component C7 (C7), complement component C9 (C9), and immunoglobulin kappa variable 1-33 (IGKV1-33). In contrast, complement component C4-B (C4B) and apolipoprotein F (APOF) exhibited either upregulation or downregulation depending on the time point.

Among the 13 proteins commonly identified in the Ta subtype across all time points, four were consistently downregulated: N-acetylmuramoyl-L-alanine amidase (PGLYRP2), carboxypeptidase N subunit 2 (CPN2), prothrombin (F2), and apolipoprotein M (APOM). In contrast, nine proteins were consistently upregulated: monocyte differentiation antigen CD14 (CD14), complement component C7 (C7), fibulin-1 (FBLN1), immunoglobulin lambda variable 3-21 (IGLV3-21), immunoglobulin heavy variable 3-49 (IGHV3-49), immunoglobulin lambda-1 light chain (X), immunoglobulin lambda variable 3-25 (IGLV3-25), apolipoprotein A-II (APOA2), and hemoglobin subunit beta (HBB).

Notably, apolipoprotein M (APOM), carboxypeptidase N subunit 2 (CPN2), and prothrombin (F2) were consistently downregulated across all patients (T1 and Ta subtypes) at all time points (*t*_0_, *t*_3_, and *t*_6_), whereas complement component C7 (C7) was consistently upregulated.

### 2.6. The Biological Role of the NMIBC-Related Proteins Identified in the PtNP–Protein Corona Before (t_0_) and After (t_3_ and t_6_) the Chemotherapy Instillation of MMC

**Figure 5:**
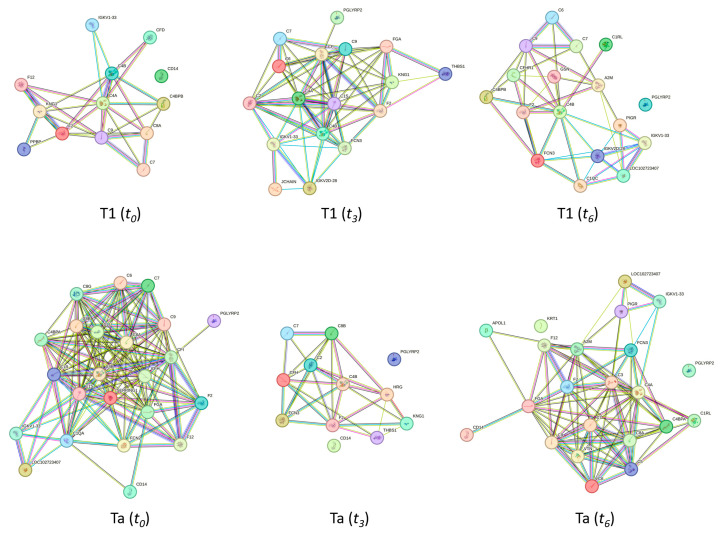
Clusters found in the protein–protein interaction network map based on the STRING database, highlighting proteins associated with the immune response pathway. At the top, clusters with 13, 17, and 17 deregulated proteins related to the immune response pathway found in the T1 subtype at *t*_0_, *t*_3_, and *t*_6_, respectively, are shown. A total of 23, 12, and 22 proteins related to the immune response pathway were deregulated in the Ta subtype at times *t*_0_, *t*_3_, and *t*_6_, respectively (down).

To analyze global changes in the serum proteome associated with the response to chemotherapy instillation of MMC in NMIBC patients with the T1 and Ta subtypes, proteins identified at the three time points were examined using the STRING software version 10.0 (see Figure 5). Consistent with the findings at time point *t*_0_, the analysis indicated that dysregulated serum proteins at *t*_3_ and *t*_6_ are primarily involved in the immune response pathway.

Figure 5 presents the clusters identified in the protein–protein interaction network map based on the STRING database, highlighting proteins associated with the immune response pathway. A total of 13, 17, and 17 proteins related to the immune response pathway were deregulated in the T1 subtype at *t*_0_, *t*_3_, and *t*_6_, respectively. However, as Figure 4 shows, 23, 12, and 22 proteins related to the immune response pathway were deregulated in the Ta subtype at times *t*_0_, *t*_3_, and *t*_6_, respectively.

Table 5 summarizes the variation in a total of 45 immune-response-related proteins during treatment for both subtypes T1 and Ta. From them, while 22 of these proteins were commonly found to be deregulated in the T1 and the Ta subtypes (C4A, C4B, C7, C8A, C9, CD14, F12, F2, IGKV1-33, KNG1, C1S, C2, C6, CFI, FCN3, FGA, PGLYRP2, THBS1, A2M, C1RL, LOC102723407, and PIGR), 8 proteins were specific to the T1 subtype (C4BPB, CFD, PPBP, IGKV2D-28, JCHAIN, C1QC, CFHR1, and GSN), and 15 proteins were specific to the Ta subtype (C1QA, C1R, C4BPA, C8B, C8G, CFH, CLU, FCN2, SERPING1, HRG, APOL1, C3, C5, KRT1, and VTN (see Figure 5 and Table 5)).

Importantly, as previously mentioned, among all proteins involved in the immune response, F2 was consistently downregulated across all patients (T1 and Ta subtypes) at all time points (*t*_0_, *t*_3_, and *t*_6_), whereas C7 was consistently upregulated.

## 3. Discussion

MMC functions by inducing DNA cross-linking, resulting in cell cycle arrest and tumor cell apoptosis [6]. The response to MMC in BC is influenced by various factors, including the TME, which plays a key role in modulating the effects of chemotherapy [34,35,36]. Among the various components of the TME, the complement system has emerged as an influential factor in regulating the immune response to cancer therapies, modulating tumor cell sensitivity to DNA-damaging agents like MMC [7].

The complement system consists of over 30 proteins that mediate a cascade of immune responses, including opsonization, inflammation, and cell lysis. These proteins are tightly regulated, and their activation can have both pro- and anti-tumor effects depending on the context [37,38,39]. Complement activation generates anaphylatoxins such as C3a and C5a, which act as chemoattractants for myeloid-derived suppressor cells (MDSCs) and tumor-associated macrophages (TAMs) [40,41]. Binding of C5a to C5aR1 on these immune cells promotes their migration into the tumor microenvironment, where they secrete immunosuppressive molecules, including reactive oxygen and nitrogen species (ROS/RNS) and interleukin-10 (IL-10). These factors attenuate CD8^+^ T cell-mediated clearance of MMC-damaged tumor cells, thereby undermining chemotherapy efficacy [42,43,44,45]. Furthermore, complement activation can influence tumor cell resistance to MMC through several mechanisms. Tumor cells often overexpress membrane complement regulatory proteins (mCRPs) such as CD55, CD46, and CD59, which inhibit complement activation and protect against complement-mediated cytotoxicity [46,47]. For instance, CD59 prevents the formation of the membrane attack complex (MAC), thereby shielding tumor cells from lysis [48,49]. Additionally, sublytic levels of MAC deposition can activate intracellular signaling pathways, including PI3K/Akt, MAPK/Erk, and NF-κB/STAT3, leading to enhanced tumor cell proliferation, survival, and upregulation of drug efflux transporters [50,51,52].

In the present work, among all deregulated complement proteins, only C7 was consistently upregulated across all patients (T1 and Ta subtypes) at all time points (*t*_0_, *t*_3_, and *t*_6_).

C7 is a critical component of the membrane attack complex (MAC). While the full MAC assembly can cause cell lysis, sub-lytic MAC deposition—facilitated by elevated C7—activates intracellular pathways in tumor cells, including Ca^2+^- and G-protein-mediated signals, and transcription factors such as EGR1, IRF1, AREG, and CXCL1. This activation enhances tumor proliferation, survival, and adaptive resistance mechanisms [53]. Furthermore, in hepatocellular carcinoma models, higher nuclear C7 expression upregulates stemness-associated genes (OCT4, SOX2, and MYC) through LSF-1 activation. These changes support tumor-initiating cell expansion, survival, and treatment resistance—including likely resistance to DNA-damaging agents like MMC [53]. Studies have also found that elevated tumor C7 expression correlates with distinct immune phenotypes and can stratify prognosis. Although more research is needed across cancer types, data indicate that higher C7 may reflect a microenvironment that favors immune suppression and resistance to therapy [7].

On the other hand, tumor-associated coagulation proteins significantly influence the response to MMC therapy by creating a protective microenvironment around cancer cells. Specifically, fibrin deposition, initiated through tumor-expressed tissue factor (TF), forms dense “fibrin clot shields” that physically impede cytotoxic immune cell binding and hinder drug penetration, thereby reducing MMC-induced tumor cell clearance [54]. Additionally, in vitro models show that these fibrin-based shields confer resistance to chemotherapeutic agents by creating a scaffold that isolates tumor cells and limits effector function of NK and LAK cells [54]. Beyond the physical barrier, coagulation proteases such as thrombin and factor Xa activate protease-activated receptors (PARs) and integrin-mediated survival signaling pathways in tumor cells, promoting PI3K/Akt and PTEN/AKT axis activation. This signaling enhances tumor proliferation, survival, and chemoresistance, and it is potentiated by fibrin–integrin β1 interactions that reinforce drug resistance phenotypes [55]. Furthermore, the cross-talk between coagulation and inflammation fosters an immunosuppressive tumor microenvironment that limits immune-mediated clearance of MMC-damaged cells and may worsen therapeutic outcome [55]. These findings suggest that elevated coagulation activity may serve as indicators of reduced MMC efficacy, while targeting coagulation pathways could enhance chemotherapy response.

In the present work, among all deregulated coagulation proteins, only F2 was consistently downregulated across all patients (T1 and Ta subtypes) at all time points (*t*_0_, *t*_3_, and *t*_6_). Tumor-associated coagulation activation drives prothrombin conversion to thrombin, which affects MMC therapy efficacy through both biological and physical mechanisms. Thrombin signals via protease-activated receptors (PAR-1 and PAR-2) on tumor, stromal, and endothelial cells, inducing oncogenic pathways such as PI3K/Akt, MAPK/Erk, and NF-κB and thereby promoting cell survival, angiogenesis, and metastasis [56,57]. Simultaneously, thrombin enhances fibrin deposition and platelet activation, contributing to a fibrin matrix “shield” around tumor cells that impedes MMC penetration and protects against immune-mediated clearance [58]. Moreover, thrombin-induced PAR activation drives an immunosuppressive microenvironment by recruiting immunosuppressive populations such as M2-like macrophages, T regulatory cells, and myeloid-derived suppressor cells via release of cytokines like IL-6, TNF-α, and MCP-1 [59,60]. This milieu diminishes immune-mediated eradication of MMC-compromised tumor cells and fosters tumor resilience.

In contrast, reduced F2 levels and, consequently, diminished thrombin activity have been shown in preclinical models to significantly impair tumor growth and survival signaling. In genetically modified mice expressing approximately 10% of normal prothrombin, subcutaneous colon adenocarcinoma growth was reduced nearly threefold compared to wild-type mice [61]. Similarly, in pancreatic ductal adenocarcinoma models (KPC2 cells), both genetic reduction and pharmacologic inhibition of prothrombin dramatically suppressed tumor mass in vivo [62]. These findings highlight that thrombin is a potent driver of tumor progression via mechanisms involving PAR-mediated signaling, angiogenesis, cellular proliferation, and metastasis [63].

Consequently, low prothrombin levels may weaken protective tumor microenvironment features such as fibrin-based shielding, thrombin-triggered survival signaling (via PAR-1 and related pathways), and immunosuppressive cell recruitment. These conditions are expected to enhance MMC delivery, attenuate DNA repair and survival pathways in tumor cells, and improve therapeutic response. Although direct clinical studies in the context of MMC are limited, the mechanistic rationale strongly suggests that patients with low circulating prothrombin levels may experience better responses to MMC by lacking the thrombin-fueled protection that typically impairs drug efficacy.

Although a direct clinical correlation between C7 levels and MMC response has not yet been well-established, measuring tumor and/or circulating C7 may help identify tumors with high sub-lytic MAC activity and stemness-associated resistance. Higher C7 levels may reflect the presence of these resistance mechanisms, predicting a reduced response to MMC therapy. Conversely, patients with low circulating F2 may experience better responses to MMC by lacking the thrombin-fueled protection that typically impairs drug efficacy. Clinically, this suggests that low F2 plus high C7 levels may inversely influence MMC response: reduced coagulation enhances MMC effectiveness, whereas elevated C7 may undermine it by activating pro-survival and stemness pathways.

Understanding the interplay between complement activation, prothrombin, and MMC offers potential therapeutic strategies to overcome resistance mechanisms in NMIBC. C7 could potentially complement other biomarkers, such as F2, to stratify patients more likely to benefit from MMC chemotherapy versus those needing alternative or combinational treatments.

Complement modulation, particularly inhibiting C7, could enhance MMC’s efficacy. Therapies that target C7’s immunosuppressive effects might reduce tumor cell survival and improve immune cell activation, thereby improving MMC’s therapeutic outcome. Combining MMC with strategies that disrupt both complement activation and coagulation may represent a promising approach to overcoming resistance to MMC and enhancing tumor cell clearance.

## 4. Materials and Methods

### 4.1. Patient Study Group and Biological Samples

Between January 2018 and June 2019, a total of 42 individuals diagnosed with NMIBC, ranging in age from 31 to 86 years, were enrolled at the University Hospital Lucus Augusti (HULA) in Lugo, Spain. An equal number of healthy individuals, matched by age, were included as controls. The NMIBC cohort was further stratified into two pathological categories: 26 patients presented with Ta stage tumors, while 16 were classified as having T1-stage disease [64,65].

In accordance with current guidelines, a single immediate postoperative intravesical instillation of a chemotherapeutic agent—specifically 40 mg MMC—after transurethral resection of the bladder tumor (TURBT) is recommended for all NMIBC patients, as numerous randomized trials and meta-analyses have shown that this strategy significantly reduces tumor recurrence (risk reduction ~25–38%) without affecting progression or survival [66]. This effect appears most pronounced in patients with solitary, small, and low-grade tumors, and benefits persist even when subsequent adjuvant intravesical therapies are administered. To maximize the efficacy of the 40 mg MMC instillation, it should be administered as promptly as possible—preferably within two hours post-TURBT, either in the recovery room or the operating theatre [67,68]. In the present study, all NMIBC patients received a single immediate intravesical instillation of 40 mg MMC following TURBT [69,70,71,72,73].

Cystoscopy at 3 months following TURBT was carried out for all NMIC patients with the Ta subtype. Patients with the T1 subtype underwent cystoscopy and urinary cytology at 3 months [74,75,76].

Peripheral venous blood samples were collected at three times: before NMIBC patients underwent surgery and/or before receiving any treatment (*t*_0_) and three months (*t*_3_) and six months (*t*_6_) after TURBT. Blood samples from the 42 age-matched HC were collected in parallel at the same timepoints.

Peripheral blood was drawn using 9 mL VACUETTE^®^ Serum Clot Activator Tubes (Greiner Bio-One, Kremsmünster, Austria). Following a 15 min clotting period at room temperature, samples were centrifuged at 1800× *g* for 5 min at 4 °C. The resulting serum was carefully aliquoted into sterile cryogenic vials, immediately frozen, and stored at −80 °C until analysis.

Ethical approval for the study was granted by the Clinical Research Ethics Committee (CEIC) of Galicia, Spain (reference number: 2017/419). All procedures were carried out in accordance with the principles outlined in the Declaration of Helsinki. Written informed consent was obtained from all participants prior to their inclusion in the study.

### 4.2. Chemicals and Reagents

All chemicals and solvents employed were of HPLC/LC-MS or electrophoresis grade. The following reagents were obtained from Merck (Barcelona, Spain): ammonium bicarbonate (AMBIC, ≥99.5%), ammonium persulfate (APS, ≥98%), β-mercaptoethanol (≥99%), glycerol (86–88%), chloroplatinic acid (H_2_PtCl_6_, ≥99%), sodium carbonate (≥99%), sodium citrate tribasic dihydrate (≥99%), tannic acid, trifluoroacetic acid (TFA, ≥99%), tris (hydroxymethyl)aminomethane (Tris-base), trizma base (≥99.9%), trypsin (from bovine pancreas), and urea. The acrylamide/bis-acrylamide solution (30%, 37.5:1) was sourced from Serva (Heidelberg, Germany). Reagents purchased from Bio-Rad (Madrid, Spain) included bromophenol blue, a Coomassie Brilliant Blue R-250 (CCB) staining solution, DL-dithiothreitol (DTT), iodoacetamide (IAA, ≥99%), sodium dodecyl sulfate (SDS), N,N,N′,N′-tetramethylethylenediamine (TEMED), and a molecular weight marker for sodium dodecyl sulfate–polyacrylamide gel electrophoresis (SDS-PAGE; range: 6.5–200 kDa). All solvents were provided by Panreac Química SLU (Barcelona, Spain).

### 4.3. Synthesis of Citrate-Coated PtNPs (2.40 ± 0.30 nm) and Ex Vivo Protein Corona Formation

**Figure 6:**
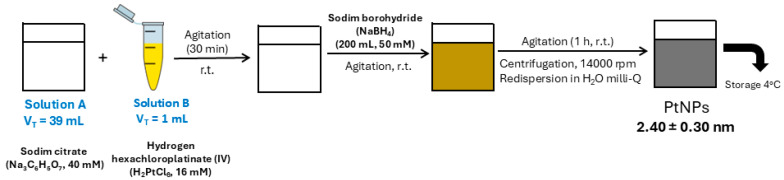
Synthesis of citrate-coated PtNPs (2.40 ± 0.30 nm).

PtNPs were synthesized using a modified version of a previously described chemical reduction protocol [22,31] (see Figure 6). Briefly, 200 µL of freshly prepared sodium borohydride (NaBH_4_, 50 mM) was added dropwise to an aqueous mixture containing 1 mL of chloroplatinic acid (H_2_PtCl_6_, 16 mM), 1 mL of trisodium citrate (40 mM), and 38 mL of deionized water. The reaction was maintained under vigorous stirring at room temperature, during which a color change to brownish-yellow signaled the formation of well-dispersed PtNPs. Stirring continued for an additional 1 h to ensure complete nanoparticle formation.

To eliminate excess trisodium citrate (SC) and sodium borohydride (NaBH_4_), the PtNPs suspension was subjected to three cycles of centrifugation at 24,610× *g* for 30 min. The purified nanoparticles were subsequently redispersed in Milli-Q water. The ex vivo formation of the PC was carried out according to the procedure outlined in Figure 2, as described in previous studies [22,28]. Characterization of the colloidal PtNPs and confirmation of PC formation were performed using TEM with a JEOL JEM-1011 instrument (Santiago de Compostela, Spain). ζ-potential measurements of the PtNPs were carried out in triplicate at 25 °C using a Malvern Zetasizer Nano ZS (Santiago de Compostela, Spain).

### 4.4. Depletion of Multiple Highly Abundant Proteins in Serum Samples

Three aliquots of human serum (30 µL each) were individually filtered through 0.22 µm Millipore Miller-GP^®^ filter units (Merck Millipore, Burlington, MA, USA) to remove particulate contaminants. Each filtered sample was then treated with 3.3 µL of freshly prepared DTT (500 mM), briefly vortexed, and incubated for 60 min to induce protein precipitation, which was evidenced by the formation of a viscous white pellet. Samples were subsequently centrifuged at 18,840× *g* for 20 min, and the resulting supernatants were collected for downstream protein alkylation and nanoparticle-assisted fractionation.

### 4.5. Isolation of Low-Abundance Proteins: Ex Vivo Protein Corona Formation

Following the depletion of high-abundance serum proteins using DTT, individual serum aliquots from each patient were processed for the enrichment and analysis of low-abundance proteins, with potential biomarker relevance. Protein alkylation was performed by incubating each aliquot with IAA at room temperature for 45 min in the absence of light to prevent degradation. Subsequently, 75 µL of PtNPs (2.40 ± 0.30 nm) was added to each sample, along with 40 µL of citrate/citric acid buffer to adjust the pH to 5.8. The mixtures were incubated at 37 °C for 30 min under continuous shaking in a thermostatic water bath to facilitate PC formation. Following incubation, nanoparticle–protein complexes were isolated via centrifugation at 24,610× *g* for 30 min. The resulting pellets were washed three times with 25 µL of citrate/citric acid buffer and centrifuged under identical conditions to remove non-specifically bound proteins. The final PC complexes associated with the PtNPs were reconstituted and separated by SDS-PAGE using PowerPac™ Basic Power Supply (Bio-Rad, Madrid, Spain). Gels were stained, and specific protein bands of interest were excised and subjected to in-gel tryptic digestion, following previously published protocols established by our group [23,25].

### 4.6. Quantification of the Proteins Presented in the Corona-Coated PtNPs by SWATH-MS

SWATH-MS acquisition and data processing were performed following a previously established protocol, with slight modifications [23]. Briefly, a spectral library was constructed using pooled serum samples representative of each study group—HC, NMIBC patients with the Ta subtype, and those with the T1 subtype—via DDA on a micro-LC system. Peptide peak extraction was carried out using the MS/MSALL add-in within PeakView Software (version 2.2, Sciex, Foster City, CA, USA), in combination with the SWATH Acquisition MicroApp (version 2.0, Sciex). Only peptides with a confidence score exceeding 99% were retained for inclusion in the spectral library.

SWATH-MS analyses were conducted on a TripleTOF^®^ 6600 LC-MS/MS instrument (Sciex) using a DIA workflow. Peak extraction and relative quantification were performed using PeakView (v. 2.2), and the resulting data were exported as mrkvw files into MarkerView software (Sciex) version 1.4 for statistical analysis. For each identified protein, the mean MS peak area across biological replicates was used for quantification. Group comparisons were conducted using a Student’s *t*-test within MarkerView based on the cumulative peak areas of all quantified transitions per protein. For protein quantitation, only peptides with a False Discovery Rate (FDR) below 1% were considered. The mean area sums of all the transitions derived for each protein in each sample will be used in a Student’s *t*-test to determine how well each variable distinguishes the two groups, which will be presented as a *p*-value. The fold change (FC) was derived from the ratio of the geometric means of the two groups, equivalently obtained by transforming data via logarithm, computing the arithmetic ratio, and then applying the inverse transformation. For each library, its set of differentially expressed proteins (*p*-value < 0.05) with an FC > 1.1 or <0.8 was selected based on previously reported methods [77,78,79,80].

### 4.7. Protein Functional Interaction Network Analysis

Analyses of functional protein interaction networks, incorporating both direct (physical) and indirect protein–protein interactions (PPIs), were performed using the STRING database (version 10.0; http://string-db.org, accessed on 15 January 2021) [81].

## 5. Conclusions

The clinical objective of this study is to identify a tumor microenvironment (TME)-associated proteomic signature that is associated with the response to mitomycin C (MMC) treatment in patients with NMIBC, specifically the Ta and T1 subtypes. To achieve this, a comparative quantitative proteomic analysis was performed on the PCs formed following the incubation of PtNPs with HS samples obtained from NMIBC patients at three distinct time points: prior to MMC instillation (*t*_0_) and at three (*t*_3_) and six (*t*_6_) months post-treatment.

This analysis revealed that many of the dysregulated serum proteins across these time points were enriched in immune-response-related pathways, underscoring the influence of systemic immune changes on therapeutic response. Notably, two proteins showed consistent differential expression across all patients and time points: F2 was consistently downregulated, while C7 was consistently upregulated in both Ta and T1 subtypes.

These findings suggest that C7 and F2 act as functionally divergent markers within the TME: Elevated C7 may drive MMC resistance through immune evasion and survival signaling, whereas reduced F2 may enhance MMC efficacy by weakening coagulation-driven tumor protection. These two proteins may therefore serve as complementary biomarkers for stratifying NMIBC patients based on likely therapeutic response. Furthermore, targeting these pathways—either by inhibiting C7-mediated complement activation or modulating thrombin-driven coagulation signaling—may provide a rational basis for combinatorial strategies aimed at overcoming resistance and improving MMC-based treatment outcomes.

Understanding this interplay between immune and coagulation systems provides valuable insight into the mechanisms underpinning MMC sensitivity and resistance in NMIBC and lays the groundwork for future therapeutic interventions aimed at reprogramming the TME to enhance clinical efficacy.

## Figures and Tables

**Table 1:** Total number of differentially expressed proteins (upregulated and downregulated; *p*-value ≤ 0.05) specific to the NMIBC subtypes (T1 and Ta) identified by SWATH-MS analysis of protein corona-coated PtNPs (2.40 ± 0.30 nm) at time point *t*_0_ (data for the T1 subtype are highlighted in blue color and for the Ta subtype in orange).

SWATH-MS Analysis						
Comparison	Protein Number (*p*-Value ≤ 0.05)					
	Total	Upregulated	Downregulated	Specific	Upregulated	Downregulated
Controls vs. T1 (*t*_0_)	39	25	14	13	4	9
Controls vs. Ta (*t*_0_)	62	53	9	36	32	4

**Table 2:** Differentially expressed proteins identified in NMIBC patients with the T1 subtype (*t*_0_) compared to HC following SWATH-MS analysis of PC-coated PtNPs (2.40 ± 0.30 nm). Proteins were classified as potential biomarkers if they exhibited a statistically significant difference (*p*-value ≤ 0.05) and a fold change (FC) greater than 1.1 (downregulated in T1 subtype: dark blue color) or less than 0.8 (upregulated in T1 subtype: light blue).

Protein Name	UniProt Name	Entry Name	Gene	*p*-Value	Fold Change
Apolipoprotein F	APOF_HUMAN	Q13790	*APOF*	0.000103989	1.665948906 ↓
Corticosteroid-binding globulin	CBG_HUMAN	P08185	*SERPINA6*	0.041311857	1.515496886 ↓
Complement C4-A	CO4A_HUMAN	P0C0L4	*C4A*	0.002227848	1.502657707 ↓
Isoform 8 of Fibronectin	FINC_HUMAN	P02751-8	*FN1*	0.039387727	1.481744201 ↓
Kininogen-1	KNG1_HUMAN	P01042	*KNG1*	0.014135446	1.458320785 ↓
Actin, alpha skeletal muscle	ACTS_HUMAN	P68133	*ACTA1*	0.041111751	1.430556317 ↓
Platelet basic protein	CXCL7_HUMAN	P02775	*PPBP*	0.025008068	1.394373864 ↓
Insulin-like growth factor-binding protein complex acid-labile subunit	ALS_HUMAN	P35858	*IGFALS*	0.021533015	1.298009336 ↓
Carboxypeptidase N catalytic chain	CBPN_HUMAN	P15169	*CPN1*	0.015062263	1.237521967 ↓
Retinol-binding protein 4	RET4_HUMAN	P02753	*RBP4*	0.027874902	0.772212849 ↑
C4b-binding protein beta chain	C4BPB_HUMAN	P20851	*C4BPB*	0.024672132	0.741162008 ↑
Complement factor D	CFAD_HUMAN	P00746	*CFD*	0.002342061	0.668694759 ↑
Beta-2-glycoprotein 1	APOH_HUMAN	P02749	*APOH*	0.004627905	0.658459944 ↑

**Table 3:** Differentially expressed proteins identified in NMIBC patients with the Ta subtype (*t*_0_) compared to HC following SWATH-MS analysis of PC-coated PtNPs (2.40 ± 0.30 nm). Proteins were classified as potential biomarkers if they exhibited a statistically significant difference (*p*-value ≤ 0.05) and an FC greater than 1.1 (downregulated in Ta subtype: dark orange color) or less than 0.8 (upregulated in Ta subtype: light orange).

Protein Name	UniProt Name	Entry Name	Gene	*p*-Value	Fold Change
Alpha-2-antiplasmin	A2AP_HUMAN	P08697	*SERPINF2*	0.001182738	1.389732364 ↓
Inter-alpha-trypsin inhibitor heavy chain H1	ITIH1_HUMAN	P19827	*ITIH1*	0.009336781	1.318100921 ↓
N-acetylmuramoyl-L-alanine amidase	PGRP2_HUMAN	Q96PD5	*PGLYRP2*	0.001225049	1.278550088 ↓
Plasma protease C1 inhibitor	IC1_HUMAN	P05155	*SERPING1*	0.021531122	1.234863507 ↓
Complement component C8 gamma chain	CO8G_HUMAN	P07360	*C8G*	0.044083487	0.829472852 ↑
Inter-alpha-trypsin inhibitor heavy chain H3	ITIH3_HUMAN	Q06033	*ITIH3*	0.029254319	0.814153389 ↑
Clusterin	CLUS_HUMAN	P10909	*CLU*	0.028873097	0.809816668 ↑
Plasma kallikrein	KLKB1_HUMAN	P03952	*KLKB1*	0.016935903	0.805823665 ↑
Complement factor I	CFAI_HUMAN	P05156	*CFI*	0.006682648	0.798543082 ↑
Complement C1r subcomponent	C1R_HUMAN	P00736	*C1R*	0.013070458	0.781890253 ↑
Fibulin-1	FBLN1_HUMAN	P23142	*FBLN1*	0.026800704	0.755595485 ↑
Complement component C8 beta chain	CO8B_HUMAN	P07358	*C8B*	0.004474085	0.754214062 ↑
Complement factor H	CFAH_HUMAN	P08603	*CFH*	0.0148658	0.73302824 ↑
Complement component C6	CO6_HUMAN	P13671	*C6*	0.001270663	0.724031596 ↑
Ficolin-2	FCN2_HUMAN	Q15485	*FCN2*	0.002023702	0.718023722 ↑
Complement C1s subcomponent	C1S_HUMAN	P09871	*C1S*	0.000559089	0.706962239 ↑
Immunoglobulin heavy variable 3-7	HV307_HUMAN	P01780	*IGHV3-7*	0.040274746	0.700272395 ↑
Fibrinogen alpha chain	FIBA_HUMAN	P02671	*FGA*	0.00059264	0.682746455 ↑
C4b-binding protein alpha chain	C4BPA_HUMAN	P04003	*C4BPA*	0.006654364	0.682409349 ↑
Vitamin D-binding protein	VTDB_HUMAN	P02774	*GC*	0.001673796	0.680683345 ↑
Complement C1q subcomponent subunit A	C1QA_HUMAN	P02745	*C1QA*	0.024744531	0.601772102 ↑
Inter-alpha-trypsin inhibitor heavy chain H4	ITIH4_HUMAN	Q14624	*ITIH4*	0.033591359	0.591696152 ↑
Apolipoprotein A-II	APOA2_HUMAN	P02652	*APOA2*	0.026952051	0.560283714 ↑
Immunoglobulin kappa variable 3-15	KV315_HUMAN	P01624	*IGKV3-15*	0.008510234	0.551467615 ↑
Immunoglobulin kappa variable 3-11	KV311_HUMAN	P04433	*IGKV3-11*	0.049717499	0.547425329 ↑
Immunoglobulin heavy variable 3-49	HV349_HUMAN	A0A0A0MS15	*IGHV3-49*	0.003041479	0.544537673 ↑
Immunoglobulin gamma-1 heavy chain	IGG1_HUMAN	P0DOX5	*-*	0.011576288	0.54073804 ↑
Immunoglobulin lambda variable 3-21	LV321_HUMAN	P80748	*IGLV3-21*	0.007386661	0.521460119 ↑
Immunoglobulin lambda variable 3-25	LV325_HUMAN	P01717	*IGLV3-25*	0.001154538	0.519943515 ↑
Immunoglobulin heavy variable 4-38-2	HVD82_HUMAN	P0DP08	*IGHV4-38-2*	0.004802612	0.499908822 ↑
Immunoglobulin lambda-1 light chain	IGL1_HUMAN	P0DOX8	*-*	0.005825155	0.46809937 ↑
Immunoglobulin kappa light chain	IGK_HUMAN	P0DOX7	*-*	0.006108806	0.461940604 ↑
Ceruloplasmin	CERU_HUMAN	P00450	*CP*	0.007023379	0.452233625 ↑
Immunoglobulin kappa variable 2-24	KV224_HUMAN	A0A0C4DH68	*IGKV2-24*	0.000204301	0.41995504 ↑
Hemoglobin subunit beta	HBB_HUMAN	P68871	*HBB*	0.008588367	0.177953493 ↑
Hemoglobin subunit alpha	HBA_HUMAN	P69905	*HBA1; HBA2*	0.007764131	0.166807835 ↑

**Table 4:** The total amount of differently expressed proteins (upregulated and downregulated, *p*-value ≤ 0.05) and those specific to different NMIBC subtypes (T1 and Ta) found after the SWATH-MS analysis of PC-coated PtNPs (2.40 ± 0.30 nm) at different times (*t*_0_, *t*_3_, and *t*_6_).

SWATH-MS Analysis							
Comparison	Protein Number (*p*-Value ≤ 0.05)						
	Total	Upregulated	Downregulated	Common	Specific	Upregulated	Downregulated
Controls vs. T1 (*t*_0_)	39	25	14	9	15	10	5
Controls vs. T1 (*t*_3_)	49	35	14		13	10	3
Controls vs. T1 (*t*_6_)	62	47	15		21	18	3
Controls vs. Ta (*t*_0_)	62	53	9	13	22	18	4
Controls vs. Ta (*t*_3_)	43	19	24		16	5	11
Controls vs. Ta (*t*_6_)	60	51	9		18	18	0

**Table 5:** Deregulated immune-response-related proteins in NMIBC patients with subtypes T1 and Ta at different time points (*t*_0_, *t*_3_, and *t*_6_). The upward-pointing arrow indicates upregulated, while the downward-pointing arrow indicates downregulated.

Immune Response Proteins							
Name	Gene	T1 Subtype			Ta Subtype		
		*t*_0_	*t*_3_	*t*_6_	*t*_0_	*t*_3_	*t*_6_
Alpha-2-macroglobulin	A2M	-	-	*↑*	-	-	↑
Apolipoprotein L1	APOL1	-	-	-	-	-	↑
Clusterin	CLU	-	-	-	↑	-	-
Coagulation factor XII	F12	↑	-	-	↑	-	↑
Complement C1q subcomponent subunit A	C1QA	-	-	-	↑	-	-
Complement C1q subcomponent subunit C	C1QC	-	-	↓	-	-	-
Complement C1r subcomponent	C1R	-	-	-	↑	-	-
Complement C1r subcomponent-like protein	C1RL	-	-	↑	-	-	↑
Complement C1s subcomponent	C1S	-	↑	-	↑	-	-
Complement C2	C2	-	↓	-	-	↓	-
Complement C3	C3	-	-	-	-	-	↑
Complement C4-A	C4A	↓	↓	-	-	-	↑
Complement C4-B	C4B	↑	↓	↓	↑	↓	-
C4b-binding protein alpha chain	C4BPA	-	-	-	↑	-	↑
C4b-binding protein beta chain	C4BPB	↑	-	↓	-	-	-
Complement C5	C5	-	-	-	-	-	↑
Complement component C6	C6	-	↑	↑	↑	-	↑
Complement component C7	C7	↑	↑	↑	↑	↑	↑
Complement component C8 alpha chain	C8A	↑	-	-	↑	-	↑
Complement component C8 beta chain	C8B	-	-	-	↑	↓	-
Complement component C8 gamma chain	C8G	-	-	-	↑	-	-
Complement component C9	C9	↑	↑	↑	↑	-	↑
Complement factor D	CFD	↑		-	-	-	-
Complement factor I	CFI	-	↑	-	↑	-	-
Complement factor H	CFH	-	-	-	↑	↓	-
Complement factor H-related protein 1	CFHR1	-	-	↑	-	-	-
Fibrinogen alpha chain	FGA	-	↑	-	↑	-	↑
Ficolin-2	FCN2	-	-	-	↑	-	-
Ficolin-3	FCN3	-	↓	↓	-	↓	↓
Gelsolin	GSN	-	-	↑	-	-	-
Immunoglobulin heavy variable 4-38-2	IGHV4-38-2	-	-	↑	↑	-	↑
Immunoglobulin J chain	JCHAIN	-	↑	-	-	-	-
Immunoglobulin kappa variable 1-33	IGKV1-33	↑	↑	↑	↑	-	↑
Immunoglobulin kappa variable 2D-28	IGKV2D-28	-	↑	↑	-	-	-
Histidine-rich glycoprotein	HRG	-	-	-	-	↑	-
Keratin, type II cytoskeletal 1	KRT1	-	-	-	-	-	↑
Kininogen-1	KNG1	↓	↓	-	-	↓	-
Monocyte differentiation antigen CD14	CD14	↑	-	-	↑	↑	↑
N-acetylmuramoyl-L-alanine amidase	PGLYRP2	-	↓	↓	↓	↓	↓
Plasma protease C1 inhibitor	SERPING1	-	-	-	↓	-	-
Platelet basic protein	PPBP	↓	-	-	-	-	-
Polymeric immunoglobulin receptor	PIGR	-	-	↑	-	-	↑
Prothrombin	F2	↓	↓	↓	↓	↓	↓
Thrombospondin-1	THBS1	-	↑	-	-	↑	-
Vitronectin	VTN	-	-	-	-	-	↑

## Data Availability

The original contributions presented in this study are included in the article/Supplementary Materials. Further inquiries can be directed to the corresponding author.

## Supplementary Materials

The supporting information can be downloaded at https://www.mdpi.com/article/10.3390/ijms26157413/s1.

## Abbreviations

CIS	Carcinoma in situ
DDA	Data-dependent acquisition
DIA	Data-independent acquisition
DLS	Dynamic light scattering
DTT	Dithiothreitol
HC	Healthy controls
HS	Human serum
IAA	Iodoacetic acid
LC-MS/MS	Liquid chromatography–tandem mass spectrometry
MDSCs	Myeloid-derived suppressor cells
MIBC	Muscle-invasive bladder cancer
MS	Mass spectrometry
NMIBC	Non-muscle invasive bladder cancer
PC	Protein corona
PPI	Protein–protein interaction
PtNPs	Platinum nanoparticles
SDS-PAGE	Sodium dodecyl sulfate–polyacrylamide gel electrophoresis
SWATH-MS	Sequential window acquisition of all theoretical mass spectra
TAMs	Tumor-associated macrophages
TEM	Transmission electron microscopy
TURBT	Transurethral resection of the bladder tumor
