# Supplementary material for: Variations in Circulating Tumor Microenvironment-Associated Proteins in Non-Muscle Invasive Bladder Cancer Induced by Mitomycin C Treatment
Source: Int J Mol Sci. 2025 Aug 1;26(15):7413. doi: 10.3390/ijms26157413 (PMC12347783; doi:10.3390/ijms26157413)
Supplement: Supplementary file 1 [file ijms-26-07413-s001.zip › ijms-3724124-supplementary.pdf]

SUPLEMENTAL MATERIAL

Table S1. TEM image and characterization data of bare PtNPs in Milli-Q-water.

PtNPs (nm)			
1	2.02	21	2.31
2	2.02	22	2.32
3	2.03	23	2.38
4	2.05	24	2.42
5	2.08	25	2.50
6	2.10	26	2.51
7	2.16	27	2.54
8	2.16	28	2.57
9	2.16	29	2.58
10	2.16	30	2.68
11	2.17	31	2.69
12	2.17	32	2.70
13	2.17	33	2.75
14	2.17	34	2.80
15	2.17	35	2.83
16	2.18	36	2.83
17	2.18	37	2.89
18	2.22	38	2.94
19	2.23	39	2.99
20	2.23	40	3.00
Count	40		
Mean	2.40		
Minimum	2.02		
Maximum	3.00		
Standar Deviation	0.30		

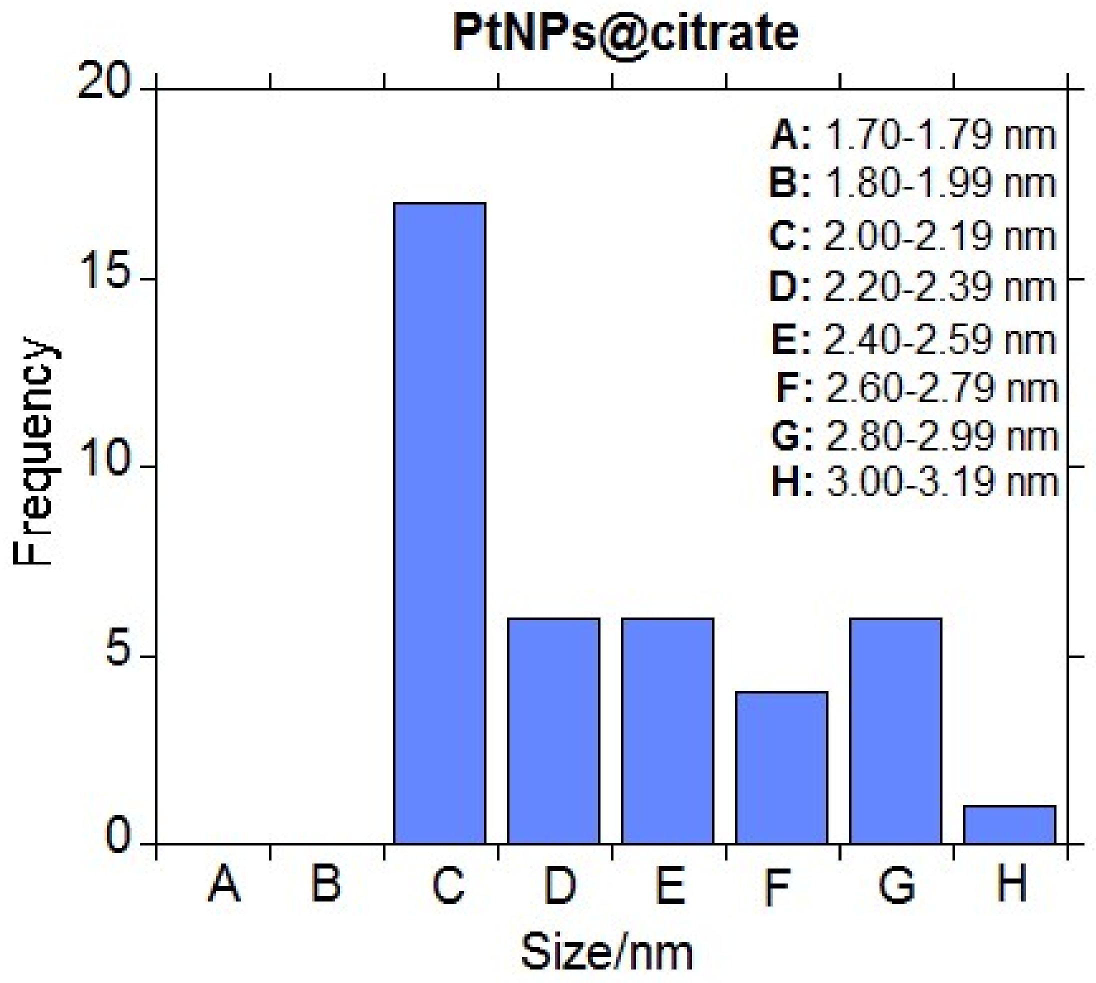
PtNPs@citrate
Frequency
Size/nm
20
15
10
5
0
A
B
C
D
E
F
G
H
A: 1.70-1.79 nm
B: 1.80-1.99 nm
C: 2.00-2.19 nm
D: 2.20-2.39 nm
E: 2.40-2.59 nm
F: 2.60-2.79 nm
G: 2.80-2.99 nm
H: 3.00-3.19 nm

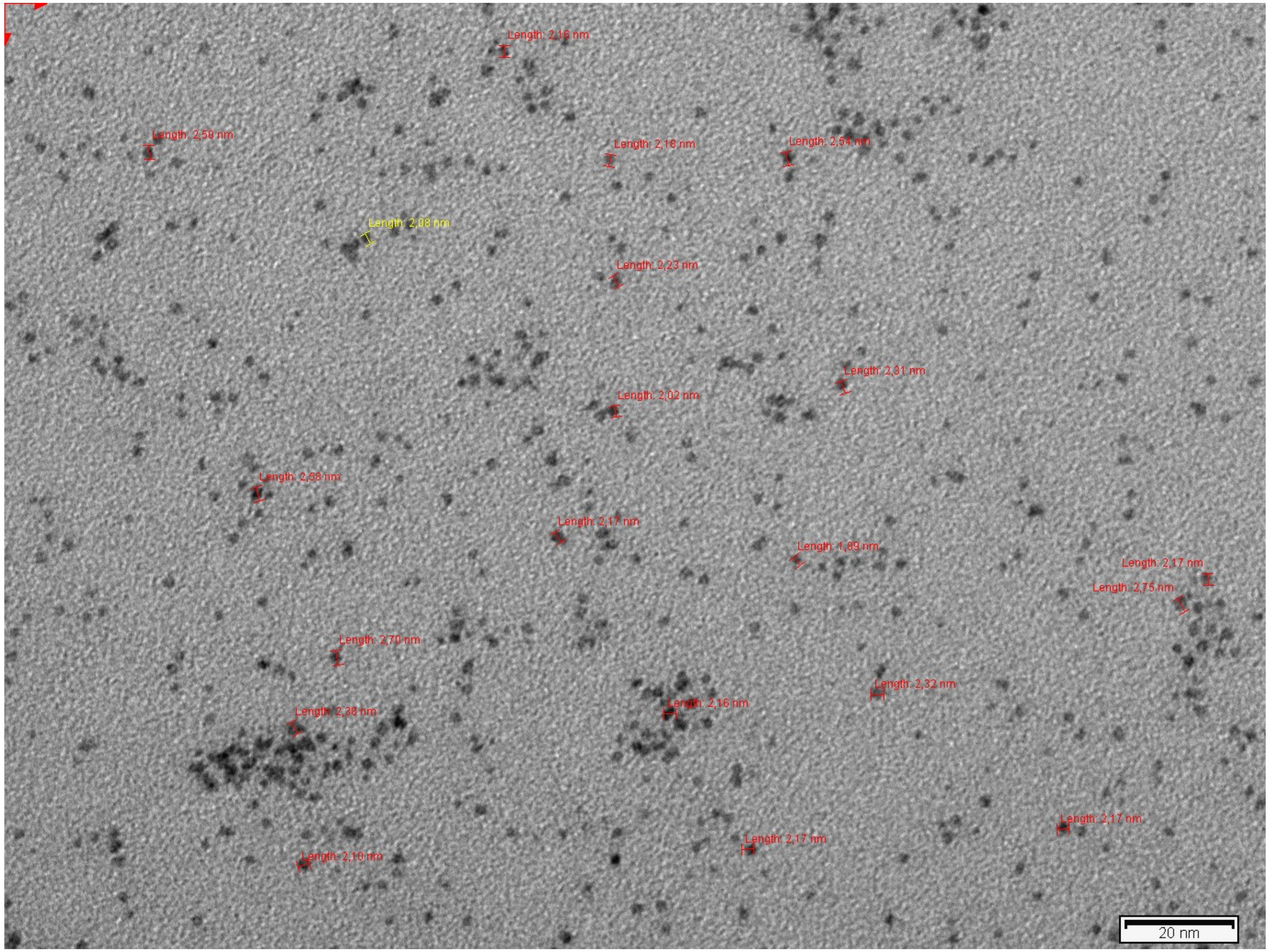
Length: 2,16 nm
Length: 2,50 nm
Length: 2,18 nm
Length: 2,54 nm
Length: 2,08 nm
Length: 2,23 nm
Length: 2,31 nm
Length: 2,02 nm
Length: 2,58 nm
Length: 2,17 nm
Length: 1,89 nm
Length: 2,17 nm
Length: 2,75 nm
Length: 2,70 nm
Length: 2,32 nm
Length: 2,16 nm
Length: 2,38 nm
Length: 2,17 nm
Length: 2,17 nm
Length: 2,10 nm
20 nm

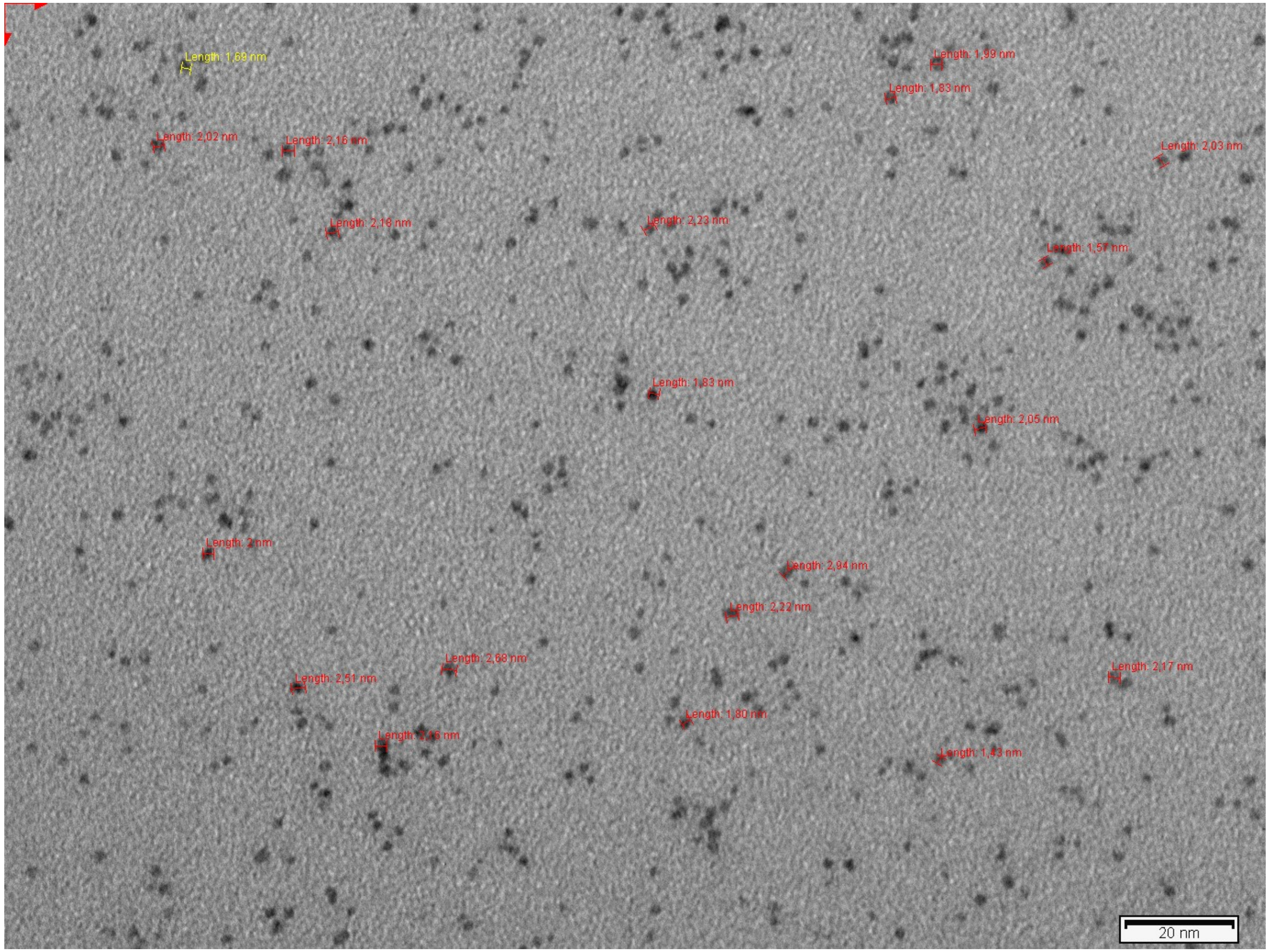
Length: 1,69 nm
Length: 1,99 nm
Length: 1,83 nm
Length: 2,02 nm
Length: 2,16 nm
Length: 2,03 nm
Length: 2,18 nm
Length: 2,23 nm
Length: 1,57 nm
Length: 1,83 nm
Length: 2,05 nm
Length: 2 nm
Length: 2,94 nm
Length: 2,22 nm
Length: 2,68 nm
Length: 2,51 nm
Length: 2,17 nm
Length: 1,80 nm
Length: 2,16 nm
Length: 1,43 nm
20 nm

Table S2. Differentially expressed proteins identified in NMIBC patients with the T1 subtype (t_0) compared to healthy controls (HC) following SWATH-MS analysis of PC-coated PtNPs (2.40 ± 0.30 nm). Proteins were classified as potential biomarkers if they exhibited a statistically significant difference (*p*-value ≤ 0.05) and a fold change (FC) greater than 1.1 (down-regulated in T1 subtype, *dark blue color*) or less than 0.8 (up-regulated in T1 subtype, *light blue*).

Protein Name	UniProt Name	Entry Name	Gene	*p*-value	Fold Change
Apolipoprotein F	APOF_HUMAN	Q13790	APOF	0.000103989	1.665948906 ↓
Apolipoprotein M	APOM_HUMAN	O95445	APOM	3.06E-06	1.588502099 ↓
Corticosteroid-binding globulin	CBG_HUMAN	P08185	SERPINA6	0.041311857	1.515496886 ↓
Complement C4-A	CO4A_HUMAN	P0C0L4	C4A	0.002227848	1.502657707 ↓
Prothrombin	THRB_HUMAN	P00734	F2	0.003711708	1.498091805 ↓
Isoform 8 of Fibronectin	FINC_HUMAN	P02751-8	FN1	0.039387727	1.481744201 ↓
Kininogen-1	KNG1_HUMAN	P01042	KNG1	0.014135446	1.458320785 ↓
Actin, alpha skeletal muscle	ACTS_HUMAN	P68133	ACTA1	0.041111751	1.430556317 ↓
Platelet basic protein	CXCL7_HUMAN	P02775	PPBP	0.025008068	1.394373864 ↓
Alpha-2-HS-glycoprotein	FETUA_HUMAN	P02765	AHSG	0.023867022	1.337194118 ↓
Serum paraoxonase/arylesterase 1	PON1_HUMAN	P27169	PON1	0.002703831	1.327437999 ↓
Insulin-like growth factor-binding protein complex acid labile subunit	ALS_HUMAN	P35858	IGFALS	0.021533015	1.298009336 ↓
Carboxypeptidase N subunit 2	CPN2_HUMAN	P22792	CPN2	0.015524051	1.254111973 ↓
Carboxypeptidase N catalytic chain	CBPN_HUMAN	P15169	CPN1	0.015062263	1.237521967 ↓
Monocyte differentiation antigen CD14	CD14_HUMAN	P08571	CD14	0.006934912	0.79335605 ↑
Galectin-3-binding protein	LG3BP_HUMAN	Q08380	LGALS3BP	0.046224882	0.785671754 ↑
Retinol-binding protein 4	RET4_HUMAN	P02753	RBP4	0.027874902	0.772212849 ↑

Complement component C7	CO7_HUMAN	P10643	C7	0.013135834	0.767794708 ↑
Coagulation factor IX	FA9_HUMAN	P00740	F9	0.001530153	0.751152223 ↑
C4b-binding protein beta chain	C4BPB_HUMAN	P20851	C4BPB	0.024672132	0.741162008 ↑
Complement component C9	CO9_HUMAN	P02748	C9	0.024269161	0.723019372 ↑
Plasminogen	PLMN_HUMAN	P00747	PLG	0.013596902	0.708631533 ↑
Platelet glycoprotein Ib alpha chain	GP1BA_HUMAN	P07359	GP1BA	0.012098377	0.669271792 ↑
Complement factor D	CFAD_HUMAN	P00746	CFD	0.002342061	0.668694759 ↑
Immunoglobulin alpha-2 heavy chain	IGA2_HUMAN	P0DOX2	-	0.032780028	0.663012168 ↑
Alpha-1B-glycoprotein	A1BG_HUMAN	P04217	A1BG	0.043941393	0.65910951 ↑
Beta-2-glycoprotein 1	APOH_HUMAN	P02749	APOH	0.004627905	0.658459944 ↑
Biotinidase	BTD_HUMAN	P43251	BTD	0.001081215	0.653546781 ↑
Complement C4-B	CO4B_HUMAN	P0C0L5	C4B	0.01904665	0.652990398 ↑
Immunoglobulin kappa variable 1-33	KV133_HUMAN	P01594	IGKV1-33	0.012179064	0.64711101 ↑
Alpha-1-antichymotrypsin	AACT_HUMAN	P01011	SERPINA3	0.04895991	0.646730692 ↑
Coagulation factor XII	FA12_HUMAN	P00748	F12	1.48E-05	0.639287522 ↑
Testis-expressed protein 33	TEX33_HUMAN	O43247	CIMIP4	0.004495746	0.629574428 ↑
Complement component C8 alpha chain	CO8A_HUMAN	P07357	C8A	0.003423768	0.607410448 ↑
Lumican	LUM_HUMAN	P51884	LUM	0.000174473	0.596350751 ↑
Immunoglobulin delta heavy chain	IGD_HUMAN	P0DOX3	-	0.036722032	0.595639641 ↑
Alpha-1-antitrypsin	A1AT_HUMAN	P01009	SERPINA1	0.048241672	0.551306835 ↑
Immunoglobulin heavy constant gamma 3	IGHG3_HUMAN	P01860	IGHG3	0.006038107	0.517525945 ↑

Serum amyloid A-4 protein	SAA4_HUMAN	P35542	SAA4	1.77E-05	0.378854495 ↑

Table S3. Differentially expressed proteins identified in NMIBC patients with the T1 subtype (t_3) compared to HC following SWATH-MS analysis of PC-coated PtNPs (2.40 ± 0.30 nm). Proteins were classified as potential biomarkers if they exhibited a statistically significant difference (*p*-value ≤ 0.05) and a FC greater than 1.1 (down-regulated in T1 subtype, *dark blue color*) or less than 0.8 (up-regulated in T1 subtype, *light blue*).

Protein Name	UniProt Name	Entry Name	Gene	*p*-value	Fold Change
Complement C4-B	CO4B_HUMAN	P0C0L5	C4B	0.001813146	1.765670628 ↓
Kininogen-1	KNG1_HUMAN	P01042	KNG1	0.003080008	1.595075108 ↓
Insulin-like growth factor-binding protein complex acid labile subunit	ALS_HUMAN	P35858	IGFALS	9.95E-06	1.514318274 ↓
Apolipoprotein E	APOE_HUMAN	P02649	APOE	0.001230222	1.474723161 ↓
N-acetylmuramoyl-L-alanine amidase	PGRP2_HUMAN	Q96PD5	PGLYRP2	0.000352544	1.438629834 ↓
Complement C2	CO2_HUMAN	P06681	C2	0.005611588	1.394598072 ↓
Complement C4-A	CO4A_HUMAN	P0C0L4	C4A	0.016540099	1.359056565 ↓
Carboxypeptidase N subunit 2	CPN2_HUMAN	P22792	CPN2	0.001376327	1.331553545 ↓
Apolipoprotein M	APOM_HUMAN	O95445	APOM	0.002381956	1.326732697 ↓
Vitamin K-dependent protein S	PROS_HUMAN	P07225	PROS1	0.001766531	1.314241562 ↓
Ficolin-3	FCN3_HUMAN	O75636	FCN3	0.008635731	1.308295789 ↓
Prothrombin	THRB_HUMAN	P00734	F2	0.010639504	1.285884784 ↓
Hyaluronan-binding protein 2	HABP2_HUMAN	Q14520	HABP2	0.024325371	1.206556647 ↓
Phosphatidylinositol-glycan-specific phospholipase D	PHLD_HUMAN	P80108	GPLD1	0.045179976	1.143819875 ↓

Complement factor I	CFAI_HUMAN	P05156	CFI	0.019330407	0.811430733 ↑
Fetuin-B	FETUB_HUMAN	Q9UGM5	FETUB	0.008261665	0.808781228 ↑
Complement component C6	CO6_HUMAN	P13671	C6	0.030831696	0.775469322 ↑
Complement component C7	CO7_HUMAN	P10643	C7	0.012623634	0.766967709 ↑
Thrombospondin-1	TSP1_HUMAN	P07996	THBS1	0.016453631	0.765903774 ↑
Retinol-binding protein 4	RET4_HUMAN	P02753	RBP4	0.009084696	0.759133672 ↑
Immunoglobulin heavy variable 3-7	HV307_HUMAN	P01780	IGHV3-7	0.042862339	0.752382211 ↑
Complement C1s subcomponent	C1S_HUMAN	P09871	C1S	0.00582828	0.743422152 ↑
Immunoglobulin J chain	IGJ_HUMAN	P01591	JCHAIN	0.02928488	0.730105279 ↑
Inter-alpha-trypsin inhibitor heavy chain H3	ITIH3_HUMAN	Q06033	ITIH3	4.01E-05	0.687734338 ↑
Complement component C9	CO9_HUMAN	P02748	C9	0.001550643	0.671118173 ↑
Fibrinogen alpha chain	FIBA_HUMAN	P02671	FGA	2.56E-05	0.6227871 ↑
Ceruloplasmin	CERU_HUMAN	P00450	CP	0.039470734	0.612579789 ↑
Galectin-3-binding protein	LG3BP_HUMAN	Q08380	LGALS3BP	0.000178784	0.563815754 ↑
Fibulin-1	FBLN1_HUMAN	P23142	FBLN1	1.28E-05	0.562298904 ↑
Immunoglobulin heavy variable 3-49	HV349_HUMAN	A0A0A0MS15	IGHV3-49	0.004901654	0.55745682 ↑
Immunoglobulin gamma-1 heavy chain	IGG1_HUMAN	P0DOX5	-	0.049454092	0.555082541 ↑
Apolipoprotein F	APOF_HUMAN	Q13790	APOF	0.000854946	0.548232003 ↑
Isoform LMW of Kininogen-1	KNG1_HUMAN	P01042-2	KNG1	0.004524806	0.537683511 ↑
Immunoglobulin kappa variable 3-15	KV315_HUMAN	P01624	IGKV3-15	0.022059904	0.526449754 ↑

Immunoglobulin kappa variable 1-33	KV133_HUMAN	P01594	IGKV1-33	0.031353837	0.525494288 ↑
Immunoglobulin lambda variable 3-21	LV321_HUMAN	P80748	IGLV3-21	0.012526106	0.516864159 ↑
Apolipoprotein C-III	APOC3_HUMAN	P02656	APOC3	0.016494668	0.505024359 ↑
Amphoterin-induced protein 2	AMGO2_HUMAN	Q86SJ2	AMIGO2	0.010996731	0.499788708 ↑
Immunoglobulin kappa variable 2D-28	KVD28_HUMAN	P01615	IGKV2D-28	0.048132488	0.474780144 ↑
Actin, alpha skeletal muscle	ACTS_HUMAN	P68133	ACTA1	0.004562944	0.469707259 ↑
Immunoglobulin kappa variable 4-1	KV401_HUMAN	P06312	IGKV4-1	0.04787217	0.467050583 ↑
Immunoglobulin lambda-1 light chain	IGL1_HUMAN	P0DOX8	-	0.017860378	0.465192934 ↑
Immunoglobulin lambda variable 8-61	LV861_HUMAN	A0A075B6I0	IGLV8-61	0.037272255	0.464821039 ↑
Immunoglobulin lambda constant 3	IGLC3_HUMAN	P0DOY3	IGLC3	0.003842815	0.417698455 ↑
Immunoglobulin heavy variable 3-23	HV323_HUMAN	P01764	IGHV3-23	0.041461774	0.408495172 ↑
Immunoglobulin kappa light chain	IGK_HUMAN	P0DOX7	-	0.007002565	0.397148719 ↑
Immunoglobulin kappa variable 3-11	KV311_HUMAN	P04433	IGKV3-11	0.00952088	0.389071143 ↑
Immunoglobulin lambda variable 3-25	LV325_HUMAN	P01717	IGLV3-25	4.78E-06	0.373658747 ↑
Apolipoprotein A-II	APOA2_HUMAN	P02652	APOA2	0.030847919	0.361429267 ↑

Table S4. Differentially expressed proteins identified in NMIBC patients with the T1 subtype (t_6) compared to HC following SWATH-MS analysis of PC-coated PtNPs (2.40 ± 0.30 nm). Proteins were classified as potential biomarkers if they exhibited a statistically significant difference (*p*-value ≤ 0.05) and a FC greater than 1.1 (down-regulated in T1 subtype, *dark blue color*) or less than 0.8 (up-regulated in T1 subtype, *light blue*).

Protein Name	UniProt Name	Entry Name	Gene	*p*-value	Fold Change
Apolipoprotein M	APOM_HUMAN	O95445	APOM	7.78E-09	1.554077235 ↓
Immunoglobulin lambda variable 1-47	LV147_HUMAN	P01700	IGLV1-47	0.013243982	1.511088279 ↓
Secreted phosphoprotein 24	SPP24_HUMAN	Q13103	SPP2	0.001479979	1.506065023 ↓
Complement C4-B	CO4B_HUMAN	P0C0L5	C4B	0.006214326	1.470100587 ↓
Insulin-like growth factor-binding protein complex acid labile subunit	ALS_HUMAN	P35858	IGFALS	7.70E-06	1.409233937 ↓
C4b-binding protein beta chain	C4BPB_HUMAN	P20851	C4BPB	0.030161799	1.391244023 ↓
Complement C1q subcomponent subunit C	C1QC_HUMAN	P02747	C1QC	0.045813116	1.320482631 ↓
Prothrombin	THRB_HUMAN	P00734	F2	0.004197145	1.279197133 ↓
Apolipoprotein F	APOF_HUMAN	Q13790	APOF	0.011278127	1.276916047 ↓
Ficolin-3	FCN3_HUMAN	O75636	FCN3	0.021470456	1.210331845 ↓
Apolipoprotein E	APOE_HUMAN	P02649	APOE	0.02119746	1.203244139 ↓
Carboxypeptidase N subunit 2	CPN2_HUMAN	P22792	CPN2	0.001089566	1.199136193 ↓
Serum paraoxonase/arylesterase 1	PON1_HUMAN	P27169	PON1	0.039209378	1.190221573 ↓
Hyaluronan-binding protein 2	HABP2_HUMAN	Q14520	HABP2	0.01683815	1.179152546 ↓
N-acetylmuramoyl-L-alanine amidase	PGRP2_HUMAN	Q96PD5	PGLYRP2	0.003151874	1.165757347 ↓

Fetuin-B	FETUB_HUMAN	Q9UGM5	FETUB	0.00336635	0.801095527 ↑
Complement component C7	CO7_HUMAN	P10643	C7	0.023936234	0.796991082 ↑
Inter-alpha-trypsin inhibitor heavy chain H1	ITIH1_HUMAN	P19827	ITIH1	0.046190246	0.792601588 ↑
Complement C1r subcomponent-like protein	C1RL_HUMAN	Q9NZP8	C1RL	0.000205466	0.786448428 ↑
Inter-alpha-trypsin inhibitor heavy chain H3	ITIH3_HUMAN	Q06033	ITIH3	0.002566719	0.783925969 ↑
Albumin	ALBU_HUMAN	P02768	ALB	0.00099122	0.771316057 ↑
Serum paraoxonase/lactonase 3	PON3_HUMAN	Q15166	PON3	0.01559954	0.767894503 ↑
Complement component C9	CO9_HUMAN	P02748	C9	0.004486636	0.745442778 ↑
Immunoglobulin alpha-2 heavy chain	IGA2_HUMAN	P0DOX2	-	0.011428584	0.730482862 ↑
Alpha-2-macroglobulin	A2MG_HUMAN	P01023	A2M	0.000378518	0.726695389 ↑
Immunoglobulin kappa variable 2D-28	KVD28_HUMAN	P01615	IGKV2D-28	0.026625568	0.722671912 ↑
Inter-alpha-trypsin inhibitor heavy chain H2	ITIH2_HUMAN	P19823	ITIH2	0.018490659	0.709586465 ↑
Alpha-1B-glycoprotein	A1BG_HUMAN	P04217	A1BG	0.038187954	0.697642961 ↑
Lumican	LUM_HUMAN	P51884	LUM	0.005623864	0.692567252 ↑
Ceruloplasmin	CERU_HUMAN	P00450	CP	0.010227074	0.683034231 ↑
Immunoglobulin heavy variable 5-10-1	HV5X1_HUMAN	A0A0J9YXX1	IGHV5-10-1	0.00104517	0.675772289 ↑
Complement component C6	CO6_HUMAN	P13671	C6	0.000704761	0.666556485 ↑
Testis-expressed protein 33	TEX33_HUMAN	O43247	CIMIP4	0.0326849	0.66491947 ↑
Hemoglobin subunit beta	HBB_HUMAN	P68871	HBB	0.015696901	0.651226615 ↑
Gelsolin	GELS_HUMAN	P06396	GSN	0.038767932	0.644912136 ↑
Biotinidase	BTD_HUMAN	P43251	BTD	0.017897769	0.634860922 ↑

Protein AMBP	AMBP_HUMAN	P02760	AMBP	2.11E-05	0.626501204 ↑
Immunoglobulin heavy variable 4-38-2	HVD82_HUMAN	P0DP08	IGHV4-38-2	0.00025626	0.626455891 ↑
Immunoglobulin kappa variable 3-15	KV315_HUMAN	P01624	IGKV3-15	0.000113546	0.626128573 ↑
Alpha-1-antichymotrypsin	AACT_HUMAN	P01011	SERPINA3	0.009901769	0.618374696 ↑
Fibulin-1	FBLN1_HUMAN	P23142	FBLN1	2.21E-06	0.61555712 ↑
Immunoglobulin kappa variable 1-33	KV133_HUMAN	P01594	IGKV1-33	0.000437525	0.610991707 ↑
Polymeric immunoglobulin receptor	PIGR_HUMAN	P01833	PIGR	0.000170888	0.600518875 ↑
Apolipoprotein A-I	APOA1_HUMAN	P02647	APOA1	0.016365911	0.592009086 ↑
Immunoglobulin heavy variable 3-23	HV323_HUMAN	P01764	IGHV3-23	0.004216426	0.588406851 ↑
Hemoglobin subunit alpha	HBA_HUMAN	P69905	HBA1; HBA2	0.004674302	0.582852215 ↑
Immunoglobulin lambda-1 light chain	IGL1_HUMAN	P0DOX8	-	9.65E-05	0.563703835 ↑
Immunoglobulin heavy variable 3-49	HV349_HUMAN	A0A0A0MS15	IGHV3-49	0.000202606	0.563379751 ↑
Immunoglobulin gamma-1 heavy chain	IGG1_HUMAN	P0DOX5	-	0.000132985	0.536772656 ↑
Immunoglobulin kappa light chain	IGK_HUMAN	P0DOX7	-	0.000705733	0.533192464 ↑
Immunoglobulin lambda variable 8-61	LV861_HUMAN	A0A075B6I0	IGLV8-61	0.027245014	0.530951809 ↑
Immunoglobulin heavy constant alpha 1	IGHA1_HUMAN	P01876	IGHA1	8.48E-06	0.522487259 ↑
Immunoglobulin kappa variable 3-11	KV311_HUMAN	P04433	IGKV3-11	0.000200701	0.507512847 ↑

Immunoglobulin lambda variable 3-25	LV325_HUMAN	P01717	IGLV3-25	2.83E-05	0.498566243 ↑
Angiotensinogen	ANGT_HUMAN	P01019	AGT	0.02620406	0.489115993 ↑
Immunoglobulin lambda variable 3-21	LV321_HUMAN	P80748	IGLV3-21	3.47E-05	0.485487535 ↑
Immunoglobulin heavy constant gamma 3	IGHG3_HUMAN	P01860	IGHG3	0.000455004	0.463999707 ↑
Immunoglobulin lambda constant 3	IGLC3_HUMAN	P0DOY3	IGLC3	3.47E-06	0.462945834 ↑
Heparin cofactor 2	HEP2_HUMAN	P05546	SERPIND1	0.024363323	0.451154258 ↑
Beta-2-glycoprotein 1	APOH_HUMAN	P02749	APOH	0.000152118	0.443694935 ↑
Complement factor H-related protein 1	FHR1_HUMAN	Q03591	CFHR1	9.15E-06	0.427292161 ↑
Amphoterin-induced protein 2	AMGO2_HUMAN	Q86SJ2	AMIGO2	5.63E-08	0.241457644 ↑

Table S5. Differentially expressed proteins identified in NMIBC patients with the Ta subtype (t_0) compared to HC following SWATH-MS analysis of PC-coated PtNPs (2.40 ± 0.30 nm). Proteins were classified as potential biomarkers if they exhibited a statistically significant difference (p-value ≤ 0.05) and a FC greater than 1.1 (down-regulated in Ta subtype, *dark orange color*) or less than 0.8 (up-regulated in Ta subtype, *light orange*).

Protein Name	UniProt Name	Entry Name	Gene	*p*-value	Fold Change
Apolipoprotein M	APOM_HUMAN	O95445	APOM	4.23E-07	1.503568986 ↓
Prothrombin	THRB_HUMAN	P00734	F2	0.000482423	1.501307322 ↓
Alpha-2-HS-glycoprotein	FETUA_HUMAN	P02765	AHSG	0.00144557	1.435848852 ↓
Alpha-2-antiplasmin	A2AP_HUMAN	P08697	SERPINF2	0.001182738	1.389732364 ↓
Inter-alpha-trypsin inhibitor heavy chain H1	ITIH1_HUMAN	P19827	ITIH1	0.009336781	1.318100921 ↓
Carboxypeptidase N subunit 2	CPN2_HUMAN	P22792	CPN2	0.001168185	1.313705993 ↓
N-acetylmuramoyl-L-alanine amidase	PGRP2_HUMAN	Q96PD5	PGLYRP2	0.001225049	1.278550088 ↓
Plasma protease C1 inhibitor	IC1_HUMAN	P05155	SERPING1	0.021531122	1.234863507 ↓
Serum paraoxonase/arylesterase 1	PON1_HUMAN	P27169	PON1	0.019526077	1.215928171 ↓
Complement component C8 gamma chain	CO8G_HUMAN	P07360	C8G	0.044083487	0.829472852 ↑
Inter-alpha-trypsin inhibitor heavy chain H3	ITIH3_HUMAN	Q06033	ITIH3	0.029254319	0.814153389 ↑
Clusterin	CLUS_HUMAN	P10909	CLU	0.028873097	0.809816668 ↑
Plasma kallikrein	KLKB1_HUMAN	P03952	KLKB1	0.016935903	0.805823665 ↑
Complement factor I	CFAI_HUMAN	P05156	CFI	0.006682648	0.798543082 ↑
Complement C1r subcomponent	C1R_HUMAN	P00736	C1R	0.013070458	0.781890253 ↑

Fibulin-1	FBLN1_HUMAN	P23142	FBLN1	0.026800704	0.755595485 ↑
Complement component C8 beta chain	CO8B_HUMAN	P07358	C8B	0.004474085	0.754214062 ↑
Monocyte differentiation antigen CD14	CD14_HUMAN	P08571	CD14	0.000443277	0.749608538 ↑
Complement factor H	CFAH_HUMAN	P08603	CFH	0.0148658	0.73302824 ↑
Complement component C6	CO6_HUMAN	P13671	C6	0.001270663	0.724031596 ↑
Ficolin-2	FCN2_HUMAN	Q15485	FCN2	0.002023702	0.718023722 ↑
Biotinidase	BTD_HUMAN	P43251	BTD	0.040666413	0.710561696 ↑
Complement C1s subcomponent	C1S_HUMAN	P09871	C1S	0.000559089	0.706962239 ↑
Immunoglobulin heavy variable 3-7	HV307_HUMAN	P01780	IGHV3-7	0.040274746	0.700272395 ↑
Platelet glycoprotein Ib alpha chain	GP1BA_HUMAN	P07359	GP1BA	0.02352854	0.692223909 ↑
Coagulation factor IX	FA9_HUMAN	P00740	F9	9.05E-05	0.688823534 ↑
Fibrinogen alpha chain	FIBA_HUMAN	P02671	FGA	0.00059264	0.682746455 ↑
C4b-binding protein alpha chain	C4BPA_HUMAN	P04003	C4BPA	0.006654364	0.682409349 ↑
Vitamin D-binding protein	VTDB_HUMAN	P02774	GC	0.001673796	0.680683345 ↑
Lumican	LUM_HUMAN	P51884	LUM	0.009804143	0.680222412 ↑
Galectin-3-binding protein	LG3BP_HUMAN	Q08380	LGALS3BP	0.013613381	0.673451469 ↑
Complement component C7	CO7_HUMAN	P10643	C7	1.22E-05	0.651402537 ↑
Plasminogen	PLMN_HUMAN	P00747	PLG	7.13E-05	0.618585178 ↑
Complement C1q subcomponent subunit A	C1QA_HUMAN	P02745	C1QA	0.024744531	0.601772102 ↑
Inter-alpha-trypsin inhibitor heavy chain H4	ITIH4_HUMAN	Q14624	ITIH4	0.033591359	0.591696152 ↑
Testis-expressed protein 33	TEX33_HUMAN	O43247	CIMIP4	0.010865462	0.584703885 ↑

Immunoglobulin heavy constant gamma 3	IGHG3_HUMAN	P01860	IGHG3	0.008689775	0.579057765 ↑
Complement component C9	CO9_HUMAN	P02748	C9	2.16E-05	0.567349589 ↑
Apolipoprotein A-II	APOA2_HUMAN	P02652	APOA2	0.026952051	0.560283714 ↑
Immunoglobulin kappa variable 3-15	KV315_HUMAN	P01624	IGKV3-15	0.008510234	0.551467615 ↑
Coagulation factor XII	FA12_HUMAN	P00748	F12	7.15E-08	0.551056907 ↑
Immunoglobulin kappa variable 3-11	KV311_HUMAN	P04433	IGKV3-11	0.049717499	0.547425329 ↑
Immunoglobulin heavy variable 3-49	HV349_HUMAN	A0A0A0MS15	IGHV3-49	0.003041479	0.544537673 ↑
Immunoglobulin gamma-1 heavy chain	IGG1_HUMAN	P0DOX5	-	0.011576288	0.54073804 ↑
Immunoglobulin alpha-2 heavy chain	IGA2_HUMAN	P0DOX2	-	0.012899886	0.528588414 ↑
Immunoglobulin lambda variable 3-21	LV321_HUMAN	P80748	IGLV3-21	0.007386661	0.521460119 ↑
Immunoglobulin lambda variable 3-25	LV325_HUMAN	P01717	IGLV3-25	0.001154538	0.519943515 ↑
Immunoglobulin delta heavy chain	IGD_HUMAN	P0DOX3	-	0.002681525	0.514284004 ↑
Immunoglobulin heavy variable 4-38-2	HVD82_HUMAN	P0DP08	IGHV4-38-2	0.004802612	0.499908822 ↑
Immunoglobulin kappa variable 1-33	KV133_HUMAN	P01594	IGKV1-33	0.002626659	0.495708869 ↑
Alpha-1B-glycoprotein	A1BG_HUMAN	P04217	A1BG	0.003712949	0.47978134 ↑
Immunoglobulin lambda-1 light chain	IGL1_HUMAN	P0DOX8	-	0.005825155	0.46809937 ↑
Immunoglobulin kappa light chain	IGK_HUMAN	P0DOX7	-	0.006108806	0.461940604 ↑

Ceruloplasmin	CERU_HUMAN	P00450	CP	0.007023379	0.452233625 ↑
Immunoglobulin kappa variable 2-24	KV224_HUMAN	A0A0C4DH68	IGKV2-24	0.000204301	0.41995504 ↑
Complement C4-B	CO4B_HUMAN	P0C0L5	C4B	1.78E-05	0.415621278 ↑
Complement component C8 alpha chain	CO8A_HUMAN	P07357	C8A	3.29E-07	0.412391461 ↑
Serum amyloid A-4 protein	SAA4_HUMAN	P35542	SAA4	0.011238051	0.253967141 ↑
Alpha-1-antitrypsin	A1AT_HUMAN	P01009	SERPINA1	0.015306147	0.203512111 ↑
Hemoglobin subunit beta	HBB_HUMAN	P68871	HBB	0.008588367	0.177953493 ↑
Hemoglobin subunit alpha	HBA_HUMAN	P69905	HBA1; HBA2	0.007764131	0.166807835 ↑
Alpha-1-antichymotrypsin	AACT_HUMAN	P01011	SERPINA3	0.011731793	0.148959389 ↑

Table S6. Differentially expressed proteins identified in NMIBC patients with the Ta subtype (t_3) compared to HC following SWATH-MS analysis of PC-coated PtNPs (2.40 ± 0.30 nm). Proteins were classified as potential biomarkers if they exhibited a statistically significant difference (p-value ≤ 0.05) and a FC greater than 1.1 (down-regulated in Ta subtype, *dark orange color*) or less than 0.8 (up-regulated in Ta subtype, *light orange*).

Protein Name	UniProt Name	Entry Name	Gene	*p*-value	Fold Change
Apolipoprotein C-I	APOC1_HUMAN	P02654	APOC1	0.0451906	2.18998679 ↓
Complement C4-B	CO4B_HUMAN	P0C0L5	C4B; C4B_2	0.000718119	1.56493034 ↓
Kininogen-1	KNG1_HUMAN	P01042	KNG1	0.000524587	1.521322657 ↓
Insulin-like growth factor-binding protein complex acid labile subunit	ALS_HUMAN	P35858	IGFALS	3.06E-08	1.469651904 ↓
N-acetylmuramoyl-L-alanine amidase	PGRP2_HUMAN	Q96PD5	PGLYRP2	3.36E-05	1.421793227 ↓
Carboxypeptidase N subunit 2	CPN2_HUMAN	P22792	CPN2	1.03E-05	1.408189179 ↓
Complement C2	CO2_HUMAN	P06681	C2	0.000160713	1.407956054 ↓
Apolipoprotein E	APOE_HUMAN	P02649	APOE	0.000254632	1.373466102 ↓
Vitamin K-dependent protein S	PROS_HUMAN	P07225	PROS1	1.26E-05	1.369745036 ↓
Hyaluronan-binding protein 2	HABP2_HUMAN	Q14520	HABP2	4.37E-06	1.36369653 ↓
Prothrombin	THRB_HUMAN	P00734	F2	0.001198317	1.302971073 ↓
Serum paraoxonase/lactonase 3	PON3_HUMAN	Q15166	PON3	0.009279709	1.269377375 ↓
Isoform 8 of Fibronectin	FINC_HUMAN	P02751-8	FN1	0.005022423	1.256039146 ↓

Serum paraoxonase/arylesterase 1	PON1_HUMAN	P27169	PON1	0.002619462	1.25350323 ↓
Secreted phosphoprotein 24	SPP24_HUMAN	Q13103	SPP2	0.030536867	1.246240118 ↓
Apolipoprotein M	APOM_HUMAN	O95445	APOM	0.003160233	1.245881552 ↓
Tetranectin	TETN_HUMAN	P05452	CLEC3B	0.036668524	1.229580526 ↓
Ficolin-3	FCN3_HUMAN	O75636	FCN3	0.017957661	1.218392267 ↓
Antithrombin-III	ANT3_HUMAN	P01008	SERPINC1	0.003291546	1.205701575 ↓
Carboxypeptidase N catalytic chain	CBPN_HUMAN	P15169	CPN1	0.03389099	1.186999629 ↓
Complement component C8 beta chain	CO8B_HUMAN	P07358	C8B	0.016967341	1.182493517 ↓
Afamin	AFAM_HUMAN	P43652	AFM	0.028141036	1.178126873 ↓
Complement factor H	CFAH_HUMAN	P08603	CFH	0.033337376	1.165357862 ↓
Coagulation factor XIII B chain	F13B_HUMAN	P05160	F13B	0.016134604	1.162366392 ↓
Monocyte differentiation antigen CD14	CD14_HUMAN	P08571	CD14	0.043754676	0.862836895 ↑
Complement component C7	CO7_HUMAN	P10643	C7	0.039014125	0.832624423 ↑
Fetuin-B	FETUB_HUMAN	Q9UGM5	FETUB	0.004638831	0.777970005 ↑
Retinol-binding protein 4	RET4_HUMAN	P02753	RBP4	0.016830443	0.756916855 ↑
Galectin-3-binding protein	LG3BP_HUMAN	Q08380	LGALS3BP	0.012106882	0.686812647 ↑
Isoform LMW of Kininogen-1	KNG1_HUMAN	P01042-2	KNG1	0.02416226	0.686048942 ↑
Fibulin-1	FBLN1_HUMAN	P23142	FBLN1	0.001348825	0.680865154 ↑
Immunoglobulin lambda variable 3-21	LV321_HUMAN	P80748	IGLV3-21	0.026203925	0.680698311 ↑
Apolipoprotein F	APOF_HUMAN	Q13790	APOF	0.002348458	0.677152871 ↑
Histidine-rich glycoprotein	HRG_HUMAN	P04196	HRG	0.03257652	0.591686552 ↑
Testis-expressed protein 33	TEX33_HUMAN	O43247	CIMIP4	0.025384088	0.586130456 ↑

Immunoglobulin heavy variable 3-49	HV349_HUMAN	A0A0A0MS15	IGHV3-49	0.01537942	0.58514523 ↑
Immunoglobulin lambda-1 light chain	IGL1_HUMAN	P0DOX8	-	0.024559515	0.554429818 ↑
Immunoglobulin lambda constant 3	IGLC3_HUMAN	P0DOY3	IGLC3	0.013357592	0.533067427 ↑
Keratin, type I cytoskeletal 9	K1C9_HUMAN	P35527	KRT9	0.014857788	0.531336773 ↑
Thrombospondin-1	TSP1_HUMAN	P07996	THBS1	0.037535948	0.514133188 ↑
Immunoglobulin lambda variable 3-25	LV325_HUMAN	P01717	IGLV3-25	0.009420566	0.500873166 ↑
Apolipoprotein A-II	APOA2_HUMAN	P02652	APOA2	0.01055576	0.435313616 ↑
Hemoglobin subunit beta	HBB_HUMAN	P68871	HBB	0.034940215	0.422165342 ↑

Table S7. Differentially expressed proteins identified in NMIBC patients with the Ta subtype (t_6) compared to HC following SWATH-MS analysis of PC-coated PtNPs (2.40 ± 0.30 nm). Proteins were classified as potential biomarkers if they exhibited a statistically significant difference (*p*-value ≤ 0.05) and a FC greater than 1.1 (down-regulated in Ta subtype, *dark orange color*) or less than 0.8 (up-regulated in Ta subtype, *light orange*).

Protein Name	UniProt Name	Entry Name	Gene	*p*-value	Fold Change
Apolipoprotein M	APOM_HUMAN	O95445	APOM	3.20E-11	1.573830881 ↓
Secreted phosphoprotein 24	SPP24_HUMAN	Q13103	SPP2	0.003608447	1.343689221 ↓
Prothrombin	THRB_HUMAN	P00734	F2	0.00023394	1.288579006 ↓
Carboxypeptidase N subunit 2	CPN2_HUMAN	P22792	CPN2	0.000161726	1.223961166 ↓
Ficolin-3	FCN3_HUMAN	O75636	FCN3	0.019586024	1.204566579 ↓
Insulin-like growth factor-binding protein complex acid labile subunit	ALS_HUMAN	P35858	IGFALS	0.006083595	1.174576179 ↓
Apolipoprotein E	APOE_HUMAN	P02649	APOE	0.046553852	1.145193289 ↓
N-acetylmuramoyl-L-alanine amidase	PGRP2_HUMAN	Q96PD5	PGLYRP2	0.046821556	1.119903855 ↓
Antithrombin-III	ANT3_HUMAN	P01008	SERPINC1	0.035833952	1.095255527 ↓
Complement C5	CO5_HUMAN	P01031	C5	0.030146779	0.878719771 ↑
Albumin	ALBU_HUMAN	P02768	ALB	0.039922602	0.858629184 ↑
Fetuin-B	FETUB_HUMAN	Q9UGM5	FETUB	0.043971129	0.854185257 ↑
Monocyte differentiation antigen CD14	CD14_HUMAN	P08571	CD14	0.008832732	0.846982365 ↑
Complement C3	CO3_HUMAN	P01024	C3	0.026265139	0.843935108 ↑
Complement C1r subcomponent-like protein	C1RL_HUMAN	Q9NZP8	C1RL	0.009007486	0.820364457 ↑

Fibrinogen alpha chain	FIBA_HUMAN	P02671	FGA	0.028986053	0.808780293 ↑
C4b-binding protein alpha chain	C4BPA_HUMAN	P04003	C4BPA	0.044796503	0.8040575 ↑
Complement C4-A	CO4A_HUMAN	P0C0L4	C4A	0.008828367	0.788590318 ↑
Vitronectin	VTNC_HUMAN	P04004	VTN	0.003843504	0.767107505 ↑
Coagulation factor XII	FA12_HUMAN	P00748	F12	0.006118999	0.757208513 ↑
Lumican	LUM_HUMAN	P51884	LUM	0.048808834	0.74726673 ↑
Complement component C8 alpha chain	CO8A_HUMAN	P07357	C8A	0.040647388	0.743135057 ↑
Immunoglobulin lambda-1 light chain	IGL1_HUMAN	P0DOX8	-	0.026633226	0.735108985 ↑
Protein AMBP	AMBP_HUMAN	P02760	AMBP	0.000998092	0.732864004 ↑
Keratin, type II cytoskeletal 1	K2C1_HUMAN	P04264	KRT1	0.005081131	0.718543814 ↑
Complement component C9	CO9_HUMAN	P02748	C9	0.000367458	0.713379132 ↑
Immunoglobulin kappa variable 1-33	KV133_HUMAN	P01594	IGKV1-33	0.017108724	0.708532735 ↑
Immunoglobulin heavy variable 5-10-1	HV5X1_HUMAN	A0A0J9YXX1	IGHV5-10-1	0.029781318	0.695171081 ↑
Ceruloplasmin	CERU_HUMAN	P00450	CP	0.009781951	0.691693068 ↑
Hemoglobin subunit beta	HBB_HUMAN	P68871	HBB	0.027520508	0.69036213 ↑
Complement component C7	CO7_HUMAN	P10643	C7	4.28E-05	0.689279263 ↑
Keratin, type I cytoskeletal 9	K1C9_HUMAN	P35527	KRT9	0.038396685	0.686662048 ↑
Complement component C6	CO6_HUMAN	P13671	C6	0.000673301	0.678609581 ↑
Alpha-2-macroglobulin	A2MG_HUMAN	P01023	A2M	0.000298629	0.672144655 ↑
Immunoglobulin heavy constant alpha 1	IGHA1_HUMAN	P01876	IGHA1	0.020509477	0.658883286 ↑
Plasminogen	PLMN_HUMAN	P00747	PLG	0.002436301	0.651368789 ↑

Immunoglobulin lambda variable 3-25	LV325_HUMAN	P01717	IGLV3-25	0.016977208	0.645475113 ↑
Apolipoprotein A-II	APOA2_HUMAN	P02652	APOA2	0.023831022	0.631292075 ↑
Immunoglobulin kappa variable 3-15	KV315_HUMAN	P01624	IGKV3-15	0.000716046	0.62246744 ↑
Immunoglobulin heavy variable 3-49	HV349_HUMAN	A0A0A0MS15	IGHV3-49	0.004631747	0.606574694 ↑
Biotinidase	BTD_HUMAN	P43251	BTD	0.006354446	0.601457279 ↑
Immunoglobulin delta heavy chain	IGD_HUMAN	P0DOX3	-	0.015075034	0.599853423 ↑
Inter-alpha-trypsin inhibitor heavy chain H4	ITIH4_HUMAN	Q14624	ITIH4	0.032737591	0.595894143 ↑
Immunoglobulin heavy variable 4-38-2	HVD82_HUMAN	P0DP08	IGHV4-38-2	0.006392954	0.591419447 ↑
Immunoglobulin lambda constant 3	IGLC3_HUMAN	P0DOY3	IGLC3	0.001857811	0.584005525 ↑
Fibulin-1	FBLN1_HUMAN	P23142	FBLN1	1.68E-05	0.574319024 ↑
Immunoglobulin alpha-2 heavy chain	IGA2_HUMAN	P0DOX2	-	0.01478654	0.571298198 ↑
Apolipoprotein A-I	APOA1_HUMAN	P02647	APOA1	0.025066041	0.560878359 ↑
Polymeric immunoglobulin receptor	PIGR_HUMAN	P01833	PIGR	0.005833412	0.559414909 ↑
Immunoglobulin gamma-1 heavy chain	IGG1_HUMAN	P0DOX5	-	0.001019703	0.55337618 ↑
Immunoglobulin heavy variable 3-23	HV323_HUMAN	P01764	IGHV3-23	0.024255762	0.547263069 ↑
Immunoglobulin lambda variable 3-21	LV321_HUMAN	P80748	IGLV3-21	0.001556691	0.542410511 ↑
Apolipoprotein L1	APOL1_HUMAN	O14791	APOL1	0.035819806	0.527275991 ↑
Alpha-1B-glycoprotein	A1BG_HUMAN	P04217	A1BG	0.018923613	0.517666973 ↑

Immunoglobulin heavy constant gamma 3	IGHG3_HUMAN	P01860	IGHG3	0.003198706	0.446527635 ↑
Heparin cofactor 2	HEP2_HUMAN	P05546	SERPIND1	0.014503051	0.367129921 ↑
Alpha-1-antichymotrypsin	AACT_HUMAN	P01011	SERPINA3	0.003651057	0.323087174 ↑
Angiotensinogen	ANGT_HUMAN	P01019	AGT	0.000370378	0.290064894 ↑
Amphoterin-induced protein 2	AMGO2_HUMAN	Q86SJ2	AMIGO2	5.53E-09	0.25576292 ↑
Alpha-1-antitrypsin	A1AT_HUMAN	P01009	SERPINA1	0.013674931	0.196226857 ↑
